# PHLI-seq: constructing and visualizing cancer genomic maps in 3D by phenotype-based high-throughput laser-aided isolation and sequencing

**DOI:** 10.1186/s13059-018-1543-9

**Published:** 2018-10-08

**Authors:** Sungsik Kim, Amos Chungwon Lee, Han-Byoel Lee, Jinhyun Kim, Yushin Jung, Han Suk Ryu, Yongju Lee, Sangwook Bae, Minju Lee, Kyungmin Lee, Ryong Nam Kim, Woong-Yang Park, Wonshik Han, Sunghoon Kwon

**Affiliations:** 1 Department of Electrical and Computer Engineering, Seoul National University, Seoul, 08826 Republic of Korea; 2 Institutes of Entrepreneurial BioConvergence, Seoul National University, Seoul, 08826 Republic of Korea; 3 Seoul National University Hospital Biomedical Research Institute, Seoul National University Hospital, Seoul, 03080 Republic of Korea; 4 Interdisciplinary Program for Bioengineering, Seoul National University, Seoul, 08826 Republic of Korea; 5 Department of Surgery, Seoul National University College of Medicine, Seoul, 03080 Republic of Korea; 6 Department of Pathology, Seoul National University College of Medicine, Seoul, 03080 Republic of Korea; 7 Cancer Research Institute, Seoul National University, Seoul, 03080 Republic of Korea; 8 Samsung Genome Institute, Samsung Medical Center, Seoul, 06351 Republic of Korea; 9 Department of Molecular Cell Biology, Sungkyunkwan University School of Medicine, Suwon, 03063 Republic of Korea

**Keywords:** Tumor heterogeneity, Cell isolation, Spatially resolved sequencing, Single-cell sequencing, Breast cancer, Precision oncology

## Abstract

Spatial mapping of genomic data to tissue context in a high-throughput and high-resolution manner has been challenging due to technical limitations. Here, we describe PHLI-seq, a novel approach that enables high-throughput isolation and genome-wide sequence analysis of single cells or small numbers of cells to construct genomic maps within cancer tissue in relation to the images or phenotypes of the cells. By applying PHLI-seq, we reveal the heterogeneity of breast cancer tissues at a high resolution and map the genomic landscape of the cells to their corresponding spatial locations and phenotypes in the 3D tumor mass.

**Electronic supplementary material:**

The online version of this article (10.1186/s13059-018-1543-9) contains supplementary material, which is available to authorized users.

## Background

Recent advances in sequencing technology have revolutionized oncology and provided an opportunity to overcome the limitations of pathological analysis [1]. However, the mutagenesis and genetic evolution in the tumor mass [2], resulting in intra-tumor heterogeneity, impede precise deciphering of a causation underlying carcinogenesis. To analyze the subclonal population in such intra-heterogeneous tumor mass, deep sequencing and computational analysis were proposed, each subclone of which is generated through subclonal expansion of progenitor tumor cells that acquired advantageous fitness after undergoing an oncogenic initial mutation [3–5]. Instead of sequencing the genomic DNA of cells from different subclones in a tumor mass separately, these approaches relied upon computational inference to separate subclones from the mixed population. Therefore, the direct relationship between the genomic data and tissue context was lost during the process. Moreover, because the heterogeneous subclones were sequenced in a pool, the detection sensitivity of variants with a low-level allele fraction was low. To overcome these limitations, other researchers adopted multi-region sequencing or laser capture microdissection instead of sequencing the whole tumor [6–8]. However, these procedures are low throughput and are usually used to process a large number of cells at once (> 1000). The ultraviolet (UV) beam used in laser capture microdissection not only hinders the throughput by burning the periphery, but also causes damage to cells [9]. Even in a microdissection using infrared (IR), which uses direct contact methods, cell debris or non-selected cells may cross-contaminate the targeted cells [10, 11]. These problems limit the use of microdissection to spatially map the heterogeneous cancer genome in a high-throughput and high-resolution manner. More recently, to provide a higher throughput and sensitivity, a single-cell analysis was proposed as a solution to thoroughly characterize the subclones and variants with a low-level allele fraction in tumors [12–15]. For example, a model of tumorigenesis and the evolutionary history of subclones in a tumor mass could be inferred by highly multiplexed single-cell genome analysis [16].

Despite the remarkable advances in sequencing technology, the foundations of modern oncology lie in histopathology, which remains the gold standard for comprehending tumorigenesis, relapse, metastasis, cancer evolution, and appropriate clinical applications [17]. Pathological assays, such as histological staining, immunohistochemistry (IHC) staining, and fluorescence in situ hybridization (FISH), directly provide the molecular information for single cells and their microenvironment. However, the conventional histopathological analyses often fail to identify subclones of cancer or rare cell types, and the subjective interpretation of the histopathological findings may lead to misunderstandings of cancer. For precise understanding of cancer and improvement in diagnostic performance, it is important to link the spatial and phenotypic histopathological information to the objective and massively parallel measurement of the genomic alteration status of cells in a high-throughput manner [18]. However, the huge amounts of sequencing data cannot be connected to the spatial and phenotypic histopathological information because the tissue-comprising cells must be pooled or dissociated in solution before sequencing. This loss of information for linking the subclonal genomic heterogeneity with the histopathological context hinders deeper analyses of the tumor mass. Spatially resolved genomics has, therefore, emerged to address this issue. Considering recent progress in spatially resolved transcriptomics [19–22], technical advances are required to map the genomic data for cancer spatially.

Here, we describe phenotype-based high-throughput laser-aided isolation and sequencing (PHLI-seq), which can efficiently detect tumor subclonality and variants with low-level allele fractions with high sensitivity and accuracy while preserving information of the 3D spatial organization and histopathological phenotypes of cells. Fully utilizing the spatial and phenotypic information [23, 24], we computationally or pathologically grouped cancer cells in tumor tissue sections into potential subclones. With the group information for the cells as guidance, cell clusters consisting of 20 to 30 neighboring cells were selected to cover all the groups and effectively represent the spatial organization of the tumor. Next, each cell cluster was physically isolated into different tubes by discharging the region of each cell cluster on an indium tin oxide (ITO)-coated glass slide using an infrared laser pulse. Finally, the extracted DNA from each tube was amplified and sequenced. The procedure, including grouping, selecting, and isolating cells, were automated with custom software to achieve a high-throughput analysis. By establishing this novel approach, we identified distinct subclones and their somatic variants in breast cancer tissues. Overall, we were able to map the heterogeneous genomic landscape of the subclones directly to the spatial and phenotypic organization of the tumor mass. We constructed genomic maps in the spatial context in two- or three-dimensional space of cancer tissues and augmented the dimension of the histopathology and genomics to understand the evolutionary histories of cancer.

## Results

### Workflow of phenotype-based high-throughput laser-aided isolation and sequencing

**Fig. 1:**
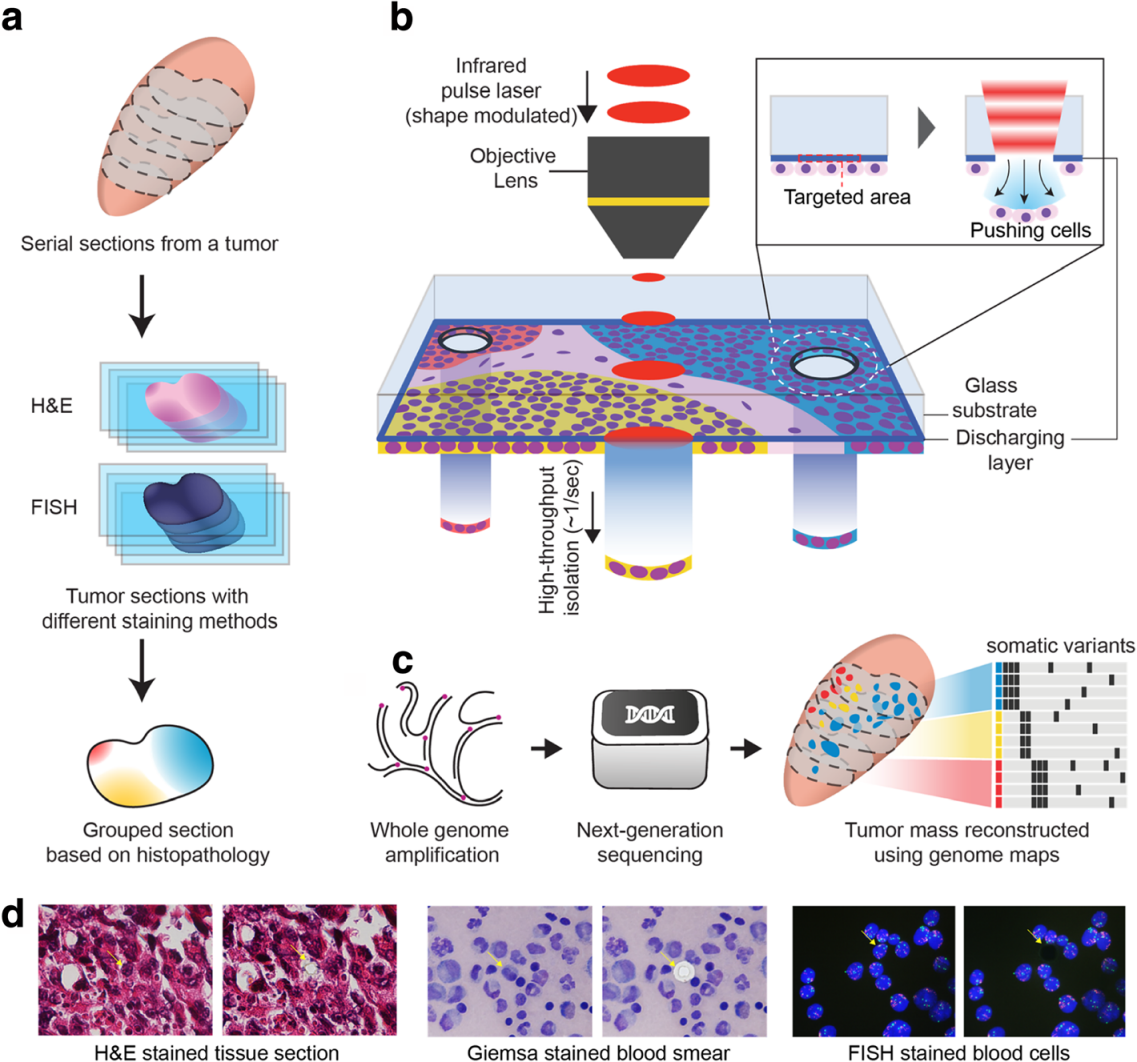
Phenotype-based high-throughput laser-aided isolation and sequencing (PHLI-seq) bridges the genotypic information to the corresponding phenotypic one in high throughput. **a** The tumor mass is sectioned and stained with H&E. The section is prepared on a discharging layer-coated glass. The tissue section is imaged, and the cancer cells in the section are grouped. Using grouping by phenotypes, cells that represent minor populations can be chosen. Grouping can be aided by pathologist manual observations. **b** A tissue section or cells prepared on the discharging layer can be isolated with an infrared (1064 nm) laser. Because the discharging layer readily vaporizes where the infrared laser is applied, the cells on the vaporized area can be released downward by pressure due to vaporization. **c** After isolating the cells, we performed whole-genome amplification and massively parallel sequencing to analyze the CNA and SNV with their spatial organization in the tissue context. **d** PHLI-seq can be applied to various types of samples, and the resolution of isolating cells can be down to single-cell level

For PHLI-seq, tissue sections or cells were stained on a transparent discharging layer-coated, or an ITO-coated, glass slide (Fig. 1a, b). To group the cells in the sample, we segmented the image of the tissue into cell clusters using a conditional random field algorithm. Then, several features, including locational information for the histopathological section and the morphological information for the cell cluster, were extracted from each of the segmented cell clusters. Finally, we generated groups based on the location and morphology of the cell clusters (Fig. 1a and the “Methods” section), and the cell clusters to be sequenced were chosen to represent all groups.

After determining the target cells, single shot of near-infrared laser (1064 nm) was applied to each targeted area of the tissue. As the near-infrared laser shot vaporized the ITO, the prepared cells on the targeted area were transferred downward by the pressure (Fig. 1b). In contrast to laser microdissection using UV cutting and catapulting [9, 25], our method, which uses an infrared laser and one-shot isolation step, causes no damage to the nucleic acids and can be performed rapidly, enabling to recover high-quality DNA from single cells or small numbers of cells in high throughput. On the other hand, standard laser microdissection using an infrared laser to heat a thermoplastic film expands and captures the cells of interest. Although this method is free of UV-induced damage, the thermoplastic film-based method requires physical contact between the film and the sample, and tearing off the cells by removing the cell-captured cap. This physical contact method causes inherent problems, such as limited throughput and scalability, debris or non-selected cell adhesion, and limited applicability depending on the sample condition [10, 11]. Using PHLI-seq, we isolated regions of cells or even single cells from various samples, regardless of how closely positioned the cells were, while preserving information regarding the spatial and histopathological phenotype context in the tissue (Fig. 1d). Together with the operating software, we were able to use PHLI-seq to isolate targets (1 s per target) in high throughput into the retrieval tubes (see Additional file 1: Note S1 and Figure S1, Additional file 2: Video S1, and Additional file 3: Video S2). When the targets were retrieved, we used multiple displacement amplification (MDA) to amplify the whole genome to high concentrations adequate for different sequencing preparation methods (i.e., whole-genome sequencing (WGS), whole-exome sequencing (WES), and targeted sequencing). After validating that the whole genome is amplified thoroughly, the high concentrations of amplicons enabled to analyze the same genome under different sequencing depths according to experimental purposes. The main purposes of the massively parallel sequencing methods were to analyze copy number alterations (CNA) and single nucleotide variants (SNV) with their spatial organization in the tissue context (Fig. 1c).


**Additional file 2:** **Video S1.** PHLI-seq video. (MP4 9094 kb)



**Additional file 3:** **Video S2.** PHLI-seq video. (MP4 19340 kb)


### Technical validation of PHLI-seq

**Fig. 2:**
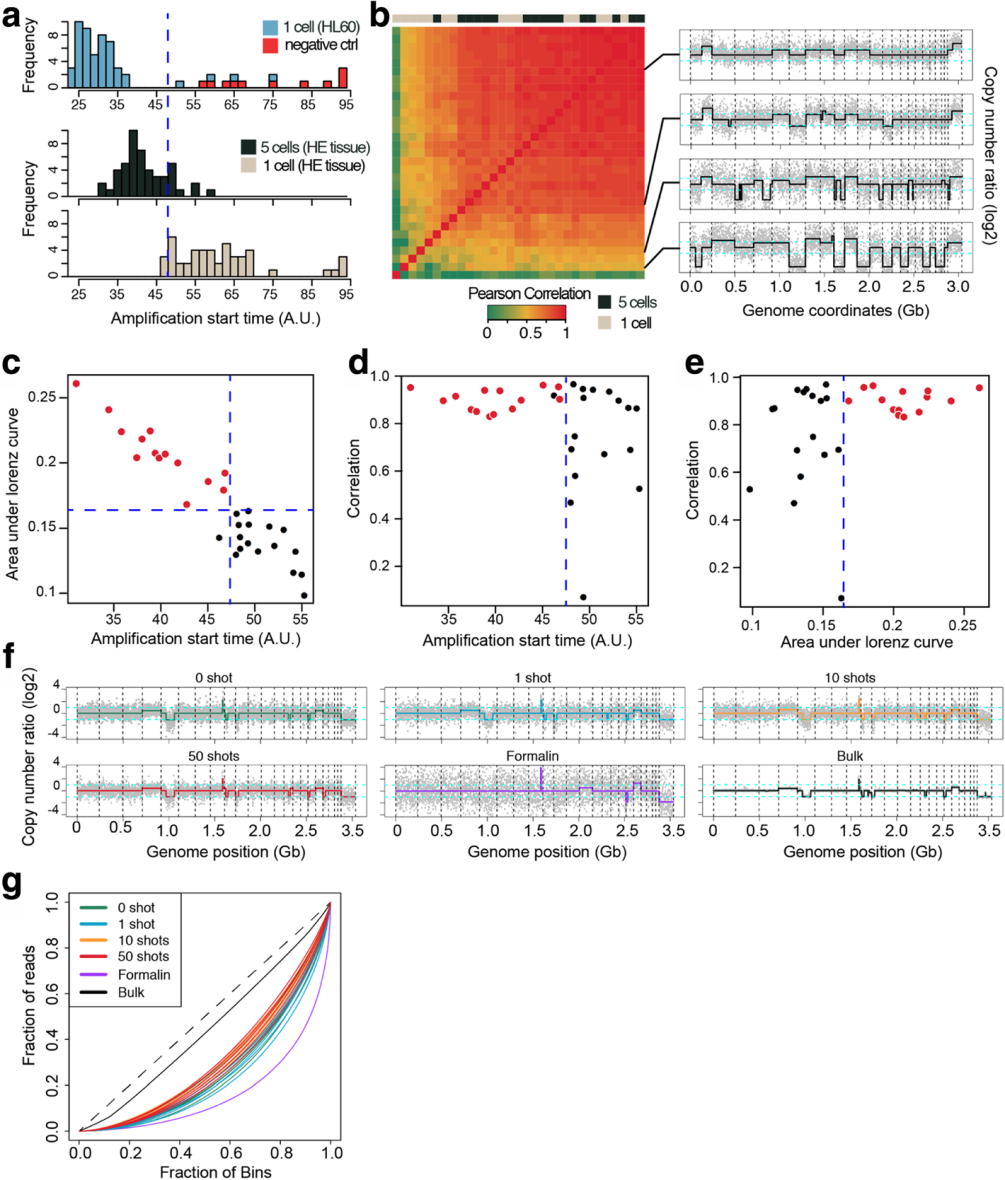
Collection efficiency and the probability of obtaining high-quality sequencing data after cell isolation by PHLI-seq. **a** HL60 cells were prepared on ITO-coated glass slide, and single cells were isolated into separate tubes (*n* = 53). MDA reaction was carried out and monitored using real-time PCR machine. After the reaction, amplification start times (AST) were determined to quantify amplification qualities for each tube. We found that negative controls or failed single-cell isolations have ASTs over 50, while successful experiments have AST under 40. Then, we performed similar experiments with an H&E-stained breast cancer tissue section. From the 10-um tissue section, five cells or single-cell were isolated and amplified with 16 replicates, respectively. A portion of the amplified products is sequenced by low-depth whole-genome sequencing. **b** To quantify amplification uniformity, we used Pearson correlations between CNA profiles of the samples and the tumor bulk DNA (left). A sample with high correlation value shows similar CNA profile with that of the tumor bulk DNA, while samples with low correlations present profiles with large deletions and variations. **c**–**e** To analyze genomic coverage, area under Lorenz curve (AUC, the higher AUC is, the higher genomic coverage is) was calculated for each sample. We found that the AUC value was inversely related to the AST. Similarly, Pearson correlation value tends to be high, when AST is low. Finally, we set a threshold value (blue dashed line) to define high-quality amplification products, which guarantees high Pearson correlation with tumor bulk DNA (i.e., amplification uniformity), and high AUC value (i.e., high genome coverage). **f**, **g** To test whether irradiating IR laser pulse and vaporizing discharging layer generate DNA fragmentation, HL60 single cells were isolated and sequenced by PHLI-seq method. Single cells were isolated by pipetting or by PHLI-seq method with 1, 10, and 50 IR laser pulse in total. Isolated cells were amplified by MDA, and six samples from each group were sequenced. For comparison, 20 cells from formalin-fixed HL60 sample and gDNA extract from a population of HL60 cells were sequenced. MDA reaction produces uniform amplification profile and high genomic coverage when input DNA is long (not fragmented) and undamaged. For example, the result from formalin-fixed sample showed low uniformity throughout the genome because the formalin fixation method is known to generate DNA fragmentation. On the other hand, the other results showed more uniform profile than that of the formalin-fixed cells. Also, the cells which were isolated by PHLI-seq technique showed similar uniformity with those isolated by pipetting, suggesting that there is no sign of laser-induced damage

To calculate the collection efficiency and the probability of obtaining high-quality sequencing data after cell isolation, we isolated single cells or very small numbers of cells from a cell line or an H&E-stained fresh-frozen cancer tissue section. The collection efficiency was 92.5% (49/53) from single-cell isolations of the cell line sample (Fig. 2a). For the experiments with H&E-stained tissue sections, we thought that nucleic acids could be damaged for amplification by MDA during preservation, tissue sectioning, and staining. Therefore, we evaluated the probability of obtaining high-quality sequencing data from 5-cell and 1-cell isolations from H&E-stained tissue sections. We found that 81.3% (13/16) and 18.8% (3/16) of the 5-cell and 1-cell isolation experiments produced high genomic coverage and correct copy number profiles (Fig. 2a). Based on the sequencing data, we also found that high genomic coverage and a high correlation with the true copy number value of the whole-genome-amplified samples were related to low *C*_T_ values for real-time monitoring of the amplification reactions (Fig. 2b–e). Therefore, we monitored the MDA procedure using a real-time PCR machine and validated the products by PCR using 16 primer panels to enrich the high-quality amplified products before sequencing (see the “Methods” section and Additional file 1: Table S1). To confirm the sequencing performance, we isolated the HL60 cells using the PHLI-seq method, sequenced their genomes, and tested the coverage breadth, allele dropout (ADO), and false-positive rate (FPR) (see Additional file 1: Note S2, Figure S2, and Figure S3). Furthermore, we tested whether the irradiating IR laser pulse and vaporizing discharging layer generate DNA fragmentations. However, we could not detect any signs of fragmentation in the sequencing data, even from cells that had repeatedly been irradiated by the IR laser pulse (Fig. 2f, g).

### Performance comparison between PHLI-seq and commercial laser microdissection technique

Commercially available laser microdissection (LMD) or laser pressure catapulting (LPC) techniques were compared to PHLI-seq. LMD technique uses UV laser to dissect a region of a sample and the polymer membrane which supports the region. LPC technique also uses UV laser but catapults a region or a cell by the pressure generated by laser-produced plasma. In using LMD or LPC, potential problems could be UV laser-induced damage, electrostatic adhesion of a dissected sample, and slow process. Therefore, we evaluated whole-genome sequencing qualities of single HL60 cells throughput LMD, LPC, and PHLI-seq technique (see the “Methods” section, Additional file 4: Video S3, Additional file 5: Video S4, and Additional file 6: Video S5).


**Additional file 4:** **Video S3.** LMD video. (MP4 16700 kb)



**Additional file 5:** **Video S4.** LPC video. (MP4 12232 kb)



**Additional file 6:** **Video S5.** PHLI-seq video. (MP4 5511 kb)


**Fig. 3:**
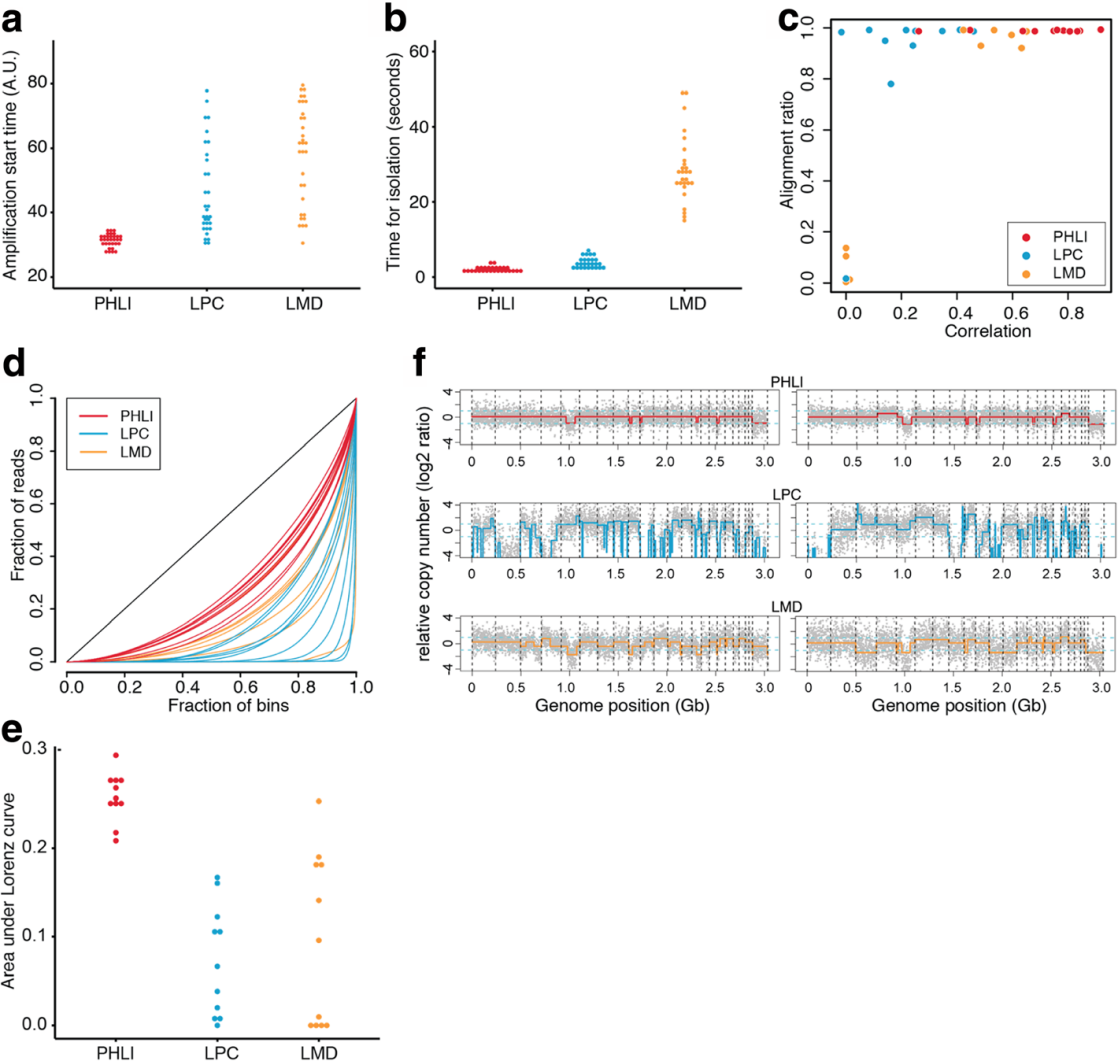
Performance comparison between PHLI-seq and commercially available laser microdissection techniques. LMD technique uses UV laser to dissect a region of a sample and the polymer membrane which supports the region. LPC technique also uses UV laser but catapults a region or a cell by pressure generated by laser-produced plasma. **a** We measured amplification start time by real-time monitoring of the MDA reactions to measure the quality of the nucleic acid in the isolated cells. **b** Also, we measured the time from targeting the cells at the computer interface to when the cells were completely isolated. These results showed that PHLI-seq outperforms commercial laser microdissection in terms of quality of DNA in isolated cells and process speed. **c** Using whole-genome sequencing data, each sample was evaluated by genome alignment ratio and copy number correlation to HL60 genome. **d**, **e** Lorenz curve provided an insight on the DNA quality after isolating cells by PHLI-seq, LPC, and LMD. This result showed that DNA quality was better when cells were isolated by PHLI-seq than LMD or LPC. **f** CNA plots of isolated single cells demonstrated that PHLI-seq could provide uniformly amplified genome with correct copy number, whereas large deletions or severe amplification bias were observed in LPC or LMD

We used a cell line rather than a tissue section, because cells in a tissue section could have partial genome by tissue sectioning, which is not suitable for a well-controlled experiment. After isolating the single cells through LMD, LPC, and PHLI-seq technique, each cell was lysed and its whole genome was amplified by MDA. Because the MDA mechanism hinders damaged or fragmented genome to be amplified thoroughly, we monitored the MDA procedure with a real-time PCR machine so that we can measure amplification start time as done with the cell line validation experiments. When we measured amplification start time by real-time monitoring of the MDA reactions to measure the quality of the nucleic acid in the isolated cells (Fig. 3a), we found that the cells isolated by PHLI-seq showed significantly earlier amplification start time with smaller deviation compared to LPC and LMD (Wilcoxon rank sum test, *p* < 10^−3^). In other words, the cells which were isolated by PHLI-seq have higher quality of nucleic acid than the cells isolated by LPC and LMD.

In addition, we measured the time from targeting the cells at the computer interface to when the cells were completely isolated. This allowed us to compare the time it takes to isolate the cells when using each technique (Fig. 3b). The result shows that LMD is much slower than other two techniques (Wilcoxon rank sum test, *p* < 10^−3^). This is because LMD must dissect the contour of the region of interest, while other techniques isolate the cells by single pulses. There was also the problem that samples could not be easily detached from the slide even after complete dissection (Additional file 4: Video S3).

The amplified DNA samples were analyzed by low-depth WGS. To get an insight on overall sequencing quality, each sample was analyzed with two metrics. The first metric was “alignment ratio,” the portion of reads aligned to the human genome. The second was “correlation,” chromosomal copy number correlation between the sequenced sample and a population of HL60 cells (Fig. 3c). From the plot, several samples that were isolated by LPC and LMD had low alignment ratio (< 0.2). Potentially, this means that a dissected or catapulted cell was not collected into a PCR tube, or isolated nucleic acid was severely damaged to be amplified. Furthermore, the single cells, which were isolated by PHLI-seq, showed higher correlations to the true HL60 genome compared to those isolated by LPC and LMD.

As aforementioned, the amplification quality can be judged by the uniformity of the amplification. In other words, the uniform amplification of a sample can indicate that the cell has been detached without damage or fragmentation (see “Formalin” in Fig. 2f, g), and the Lorenz curve could provide an insight on the DNA quality after isolating cells by PHLI-seq, LPC, and LMD (Fig. 3d, e). This result shows that the DNA quality was highest when cells were isolated by PHLI-seq. Then, we selected two highest quality samples from each isolation method and presented their genome-wide copy numbers (Fig. 3f). The CNA plots from PHLI-seq technique showed uniformly amplified genome with correct copy number except for single-cell variability. In contrast, the CNA plots from LPC and LMD presented large deletion or severe amplification bias.

### Applying PHLI-seq to hormone receptor-positive/human epidermal growth factor receptor 2-positive breast cancer

Next, we applied PHLI-seq to a hormone receptor (HR)-positive/human epidermal growth factor receptor 2 (HER2)-positive invasive ductal carcinoma (IDC) to demonstrate that the genomes of the cell clusters from a stained tissue section can be effectively analyzed. Based on an image analysis and inspection by pathologists, 53 cell clusters were selected in the tissue section for analysis by PHLI-seq (see Additional file 1: Note S3 and Figure S4a-d). With an average of 20–30 cells in each cell cluster, whole-genome amplification and quality filtering were performed (see the “Methods” section). The filter-passed samples were analyzed by low-depth whole genome (*n* = 53), whole exome (*n* = 12), and targeted sequencing (*n* = 53) (see the “Methods” section).

**Fig. 4:**
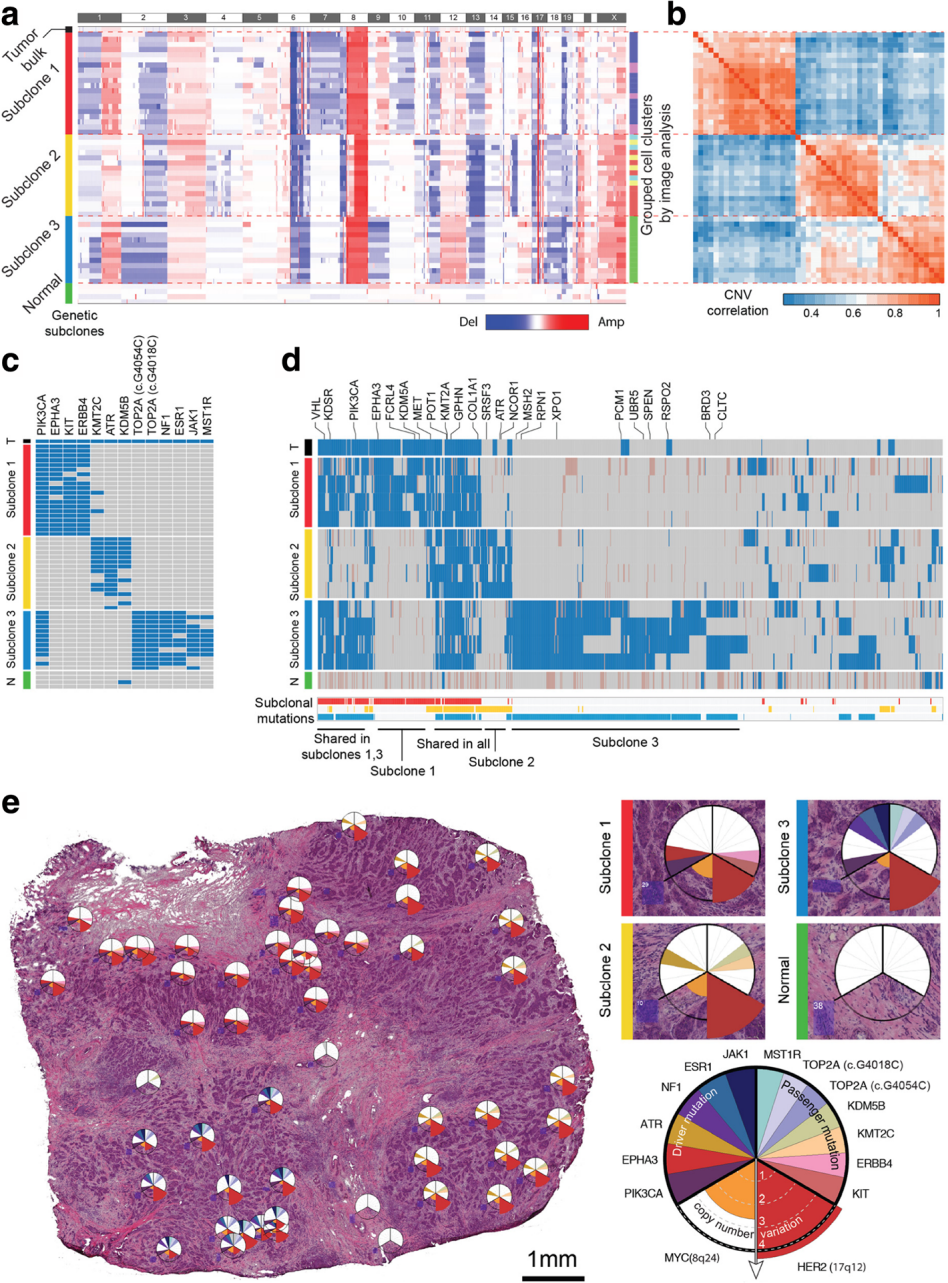
CNA and SNV analyses by whole-genome, targeted, and whole-exome sequencing and its validation by single-molecule deep sequencing reveal three different subclones of the cancer tissue. **a** After performing low-depth whole-genome sequencing (0.16 Gb/sample), CNA was determined as described in the “Methods” section. Rows were reordered to cluster samples by the correlation of their CNAs. Excluding the tumor and normal bulk, we found three distinct genetic subclones. The three subclones had both shared and exclusive CNA events. **b** The correlation matrix of the copy number data also produced the three subclones. Unsupervised clustering was used for reordering of the samples. **c** Targeted sequencing of the 53 isolated cell clusters. As in the CNA analysis, we found three distinct genetic subclones. The three subclones had unique somatic mutation patterns. **d** Whole-exome sequencing of 4 samples from each subclone. Subclones 1 and 3 shared a large portion of mutations, including the *PIK3CA* mutation. **e** Spatial mapping of genomic data showing that each subclone is spatially segregated, with stroma between each subclone

First, genome-wide CNA analysis by low-depth whole-genome sequencing revealed three major subclonal populations in the tumor sample (Fig. 4a, b, approximate unbiased *p* value > 0.99, multiscale bootstrap resampling with 10,000 iterations, see the “Methods” section). The three subclonal populations had both shared and unique alteration profiles. The shared alterations include 1q gain, 8q gain, 8p loss, and HER2 amplifications, all of which had been previously reported as frequent CNAs in human breast cancer and other types of cancer [26, 27]. One interesting observation is that the CNA status was clearly divided into three distinct populations with no intermediate subclones. Since intermediate subclones might be excluded from the sampling process, we isolated additional cell clusters (*n* = 27) at the boundaries between subclones. The isolated samples were analyzed by low-depth whole-genome sequencing. Then, clustering analysis was performed based on the inferred copy number data for both previously isolated (*n* = 53) and additionally isolated samples (*n* = 27). The results showed that the 80 cell clusters from the HER2-positive tissue sections were classified into one of the three previously defined cancer subclones (see Additional file 1: Figure S5). This result reinforces the evidence for the punctuated copy number change followed by a period of stasis, as demonstrated in previous studies [16, 18, 28].

To investigate somatic SNV, we performed targeted sequencing of 121 genes associated with breast cancer (see the “Methods” section and Additional file 1: Table S2). The results revealed unique mutational profiles in each subclone, consistent with those determined by whole-genome sequencing (Fig. 4c). In our targeted sequencing analysis of 53 cell cluster samples, we found that mutations in *PIK3CA*, *EPHA3*, *KIT*, *ERBB4*, and *KMT2C* occurred in subclone 1; mutations in *KMT2C*, *ATR*, and *KDM5B* in subclone 2; and mutations in *TOP2A*, *NF1*, *ESR1*, *JAK1*, and *MST1R* in subclone 3. For further analysis, we performed whole-exome sequencing of four samples selected from each subclone (Fig. 4d). We found that 75 mutations were shared in the three subclones and that 99, 75, and 382 mutations in *VHL*, *KDSR*, *PIK3CA*, *EPHA3*, *FCRL4*, *KDM5A*, *MET*, *POT1*, *KMT2A*, *GPHN*, *COL1A1*, *SRSF3*, *ATR*, *NCOR1*, *MSH2*, *RPN1*, and *XPO1* occurred exclusively in subclones 1, 2, and 3, respectively. In contrast to the whole-exome mutation profiles in the three subclones by PHLI-seq, we could not find such representative mutation profiles in the sequencing data from the tumor bulk. This result implies that PHLI-seq can provide rich information about subclonality and variants with a low-level allele fraction in heterogeneous tumors, even those with subclones that are too minor to be detected by conventional methods.

Based on the CNA and SNV analysis, we inferred the evolutionary history of the subclones in the tumor (see Additional file 1: Note S3 and Figure S6). Also, we mapped the detailed information for the CNAs, driver mutations, and passenger mutations to the topological information and spatial positions of the tumor tissue (Fig. 4e). The three subclones were found to be spatially segregated in the tumor mass. As shown in Fig. 4e, whereas the heterogeneity of the tumor tissue is clear from the detection of the three different subclones, the micro area occupied by each subclone exhibits no mingling with cells from other subclones. This finding implies that the three subclones are independent with well-established tumorigenic advantages and strongly suggests that a combination of diverse drugs for inhibiting different subclones in each patient should be a future therapeutic strategy for personalized cancer medicine.

### Constructing and visualizing a cancer genomic map in a three-dimensional spatial context

**Fig. 5:**
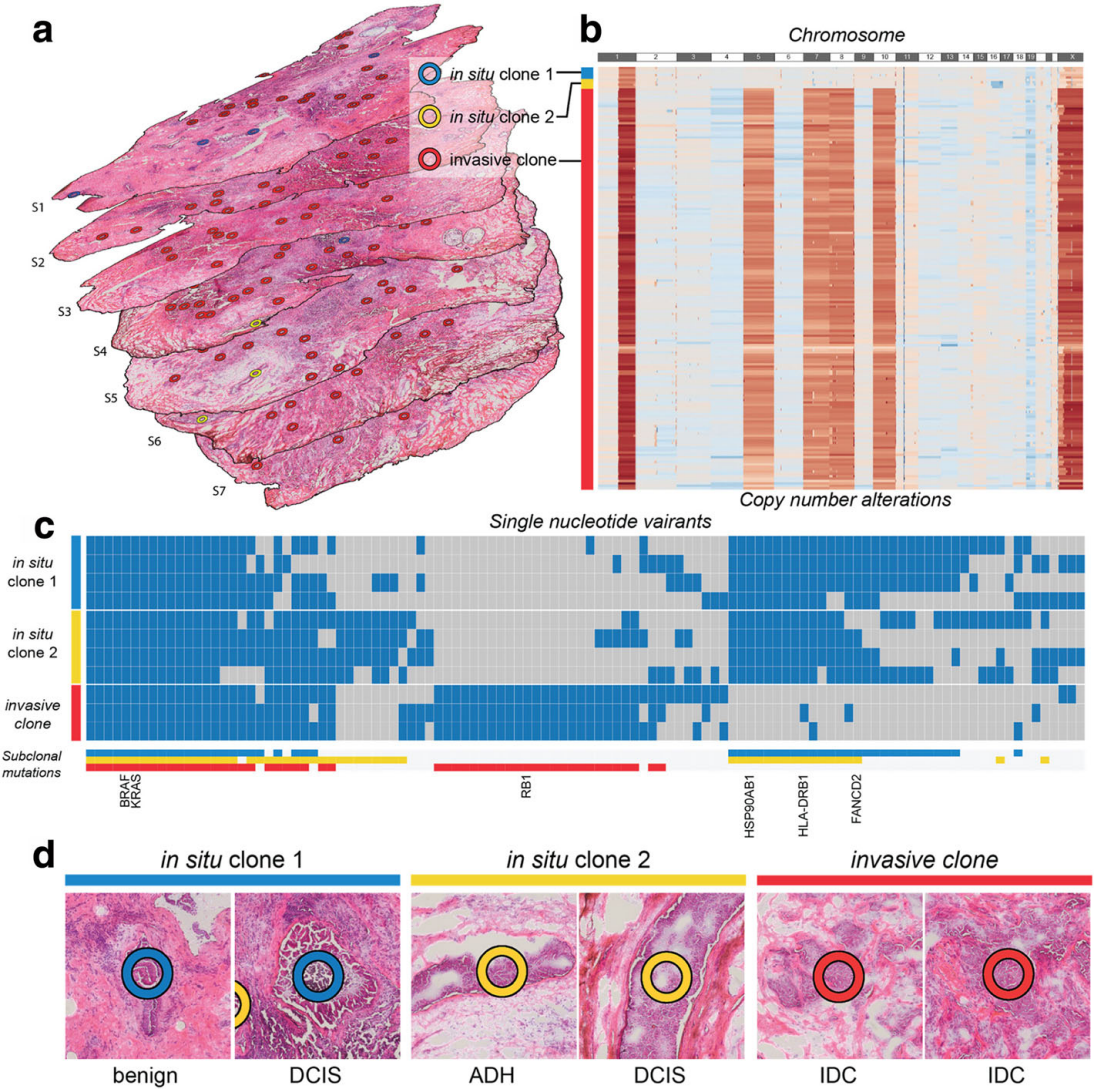
The three-dimensional tumor mass was investigated using PHLI-seq. **a** A total of 177 cell clusters were isolated and analyzed from 7 consecutive tissue sections from a triple-negative breast tumor. Before the isolation, cancer cells with various phenotypes were identified by histopathological evaluation from H&E and IHC (AR, CK5/6, Ki-67, and p53; see Additional file 1: Figure S7) sections. Based on the phenotypic information, the 177 cell clusters were selected to discover genetic heterogeneity in tumor cells with various phenotypes. Seven consecutive tissue sections had a 700-μm interval between each section. **b** Whole-genome sequencing of the 177 cell clusters in the tumor discovered three subclones. The in situ clones 1 and 2 shared CNAs in chromosomes 1 and X. On the other hand, the invasive clone had considerably more amplifications in chromosomes 1 and X, and additional amplifications in chromosomes 5, 7, 8, and 10. **c** Whole-exome sequencing of the selected samples from each subclone. The subclonal mutations are labeled in the corresponding color to demonstrate shared and unique mutations. **d** In situ clone 1 included DCIS and benign usual ductal hyperplasia, whereas in situ clone 2 included DCIS and ADH. Histopathologic evaluation of the invasive clone showed that every cell clusters in this subclone were IDC

We further analyzed consecutive sections of a triple-negative (estrogen/progesterone receptor and HER2-negative) breast cancer sample to discover how heterogeneous tumor subclones exist in the three-dimensional space of the tissue and to demonstrate how PHLI-seq can be an empowering tool to bridge genomics to histopathology (Fig. 5a). The size of the tumor was about 7 × 6 × 5 mm, and seven tissue slices with an interval of 700 μm between each of them were used to prepare H&E sections for PHLI-seq. A total of 177 cell clusters from the seven H&E sections were isolated and sequenced by PHLI-seq as described. Before the isolation, cancer cells with various phenotypes were identified by histopathological evaluation from H&E and IHC (AR, CK5/6, Ki-67, and p53; see Additional file 1: Figure S7) sections. Based on the phenotypic information, the 177 cell clusters were selected to discover genetic heterogeneity in tumor cells with various phenotypes. After isolating the targeted cell clusters, we performed low-depth whole-genome sequencing to obtain an image of the heterogeneity of CNAs (Fig. 5b). We discovered three genetic subclones, and they were denoted in situ clone 1, in situ clone 2, and invasive clone. In situ clone 1 and in situ clone 2 had the same copy number profiles, except for the deletion of a q arm of chromosome 16 and a p arm of chromosome 17 in in situ clone 2, suggesting that the in situ clone 2 may have derived from ancestral cells of the in situ clone 1 by additional chromosomal deletions. The invasive clone showed additional chromosomal amplification in chromosomes 1 (q arm), 6, 7, 8, 10, and X. To investigate more genomic differences at the single nucleotide level, we performed whole-exome sequencing (WES) for 11 clusters in the three clones (Fig. 5c). The result shows that the tumor cells in the invasive clone had mutations which do not exist in in situ clones. Surprisingly, even in situ clones had mutations which were not observed in the invasive clone. This may suggest that IDC is derived from an early ancestry of ductal carcinoma in situ (DCIS), atypical ductal hyperplasia (ADH), or other benign cells, not directly from DCIS in a linear manner. Moreover, in situ clone 2 showed mutations exclusive to in situ clone 1, which supports the previous explanation that in situ clone 2 may be derived from the ancestral cells of in situ clone 1, based on CNA profiles.

Within the two in situ clones determined by whole-genome sequencing, cells with different histopathologic features were observed. DCIS and benign usual ductal hyperplasia were included in in situ clone 1, whereas DCIS and ADH were included in in situ clone 2. Histopathologic evaluation of the invasive clone showed that every cell cluster in the invasive clone was IDC (Fig. 5d). A three-dimensional reconstruction of the sections allowed identification of in situ clone 2 clusters observed in a vicinity in consecutive sections S4, S5, S7, and S7, suggesting that combined analysis of histopathology and spatially resolved genomics enabled by PHLI-seq has potential to contribute to clinical diagnostics.

## Discussion

In this study, we developed a new technology that effectively discovers the genetic heterogeneity of tumors with accurate mapping of genomic alterations to the spatial information of the tissues. We showed that PHLI-seq technique outperformed conventional UV laser microdissection technique in terms of DNA quality and sequencing result of isolated cells and process speed (Fig. 3 and Additional file 1: Figure S12). This could result from wavelength (355 nm) and applied energy (~ 10 μJ) of the laser used in LPC and LMD. We used 1064 nm of IR laser for PHLI-seq, and photons in this wavelength have three times lower energy compared to the UV laser in LPC and LMD. Moreover, we used ~ 3 μJ of single laser pulse for PHLI-seq, whereas LPC and LMD used about three times higher energy per a laser pulse. Especially in LMD, thousands of laser pulses were irradiated around sample for dissection (80 Hz laser pulse × 30 s = 2,400 laser pulses). In addition to UV-induced damage of laser microdissection, high equipment costs can prevent this technique from being widely adopted for genome research. Although basic optic and mechanical components are similar in PHLI-seq and laser microdissection, replacing UV laser optics (lenses or mirrors which can withstand UV damage and correct chromatic aberration) can reduce manufacturing cost. Therefore, we believe that PHLI-seq is advantageous both in performance and cost.

Using PHLI-seq, we identified three subclones in the HR-positive/HER2-positive breast cancer tissue and discovered subclonal CNAs and point mutations. Here, we found that the majority of CNAs are shared by all groups, whereas those of SNVs are not. Similar to a prior report [12], this result indicates that most chromosomal rearrangements preceded the generation of point mutations. Moreover, when only CNAs are considered, the evolution of the tumor seems to have followed the model of punctuated evolution with a one-time event, rather than the model of gradual evolution [16, 18]. However, regarding the SNVs, the evolution cannot be explained by the model of punctuated evolution with a one-time event. Because subclones 1 and 3 share many SNVs, the divergence of subclones 1 and 3 from the ancestral population seems to have occurred later than the evolutionary burst that generated subclone 2 and the ancestral population of subclones 1 and 3. Therefore, considering both the CNAs and the SNVs, our data can be explained by the model of punctuated copy number evolution with two-time events. Overall, we visualized the three genetic subclones of the HR-positive/HER2-positive breast cancer in tissue context with their genetic history of punctuated copy number evolution.

We also applied PHLI-seq to serial breast cancer tissue sections to construct and visualize a cancer genomic map in a three-dimensional spatial context (see Additional file 7: Video S6). From 177 cell clusters in a 7 mm × 6 mm × 5 mm space, we identified three genetic subclones encompassing the histopathologically classified benign cancer cells ADH, DCIS, and IDC. We performed CNA and SNV analyses by WES to elucidate the evolutionary history of the cancer cells in relation to the histopathological information. In three-dimensional space, the invasive clone was located throughout the entire tissue, while the in situ clones were confined to relatively smaller areas. Although in situ clone 2 clusters were observed in a vicinity in four consecutive sections, it is interesting that the in situ clone 1 was observed in sections S1 and S4, but not in between them (Fig. 5a). Despite thorough observation of sections S2 and S3, we could not detect any benign cells or DCIS in the histopathological evaluation. Moreover, we could not detect exact spatial linkage between in situ clones 1 (sections S1 and S4) and 2 (sections S4, S5, S6, and S7). One explanation could be that the invasiveness of tumor cells in the invasive clone resulted in the spatial separation of the in situ clones, especially between the in situ clone 1 cell clusters in sections S1 and S4. This suggests that different tumors show various and complex spatial context, and PHLI-seq is the only method that allows for correlated analysis of sequencing data and histopathological spatial features.


**Additional file 7:** **Video S6.** 3D reconstruction video. (MP4 14255 kb)


This new high-throughput technology can accurately bridge the histopathological and genomic alteration landscape by leveraging the map of genomic alteration at the single-cell level to spatial positions in a tissue, allowing an unprecedented better understanding of carcinogenesis. Additionally, by comparing primary cancers with metastatic or recurrent cancers by high-precision analysis of micro-local tissue areas at the single-cell level using PHLI-seq, one can discover novel important molecular features concerning the carcinogenic transition between different tumorigenic stages. In addition, this technology can provide novel insights at the high-precision single-cell level of interactions between a tumor cell and its microenvironment, which has not been clearly elucidated by previous conventional techniques.

Another new innovative improvement supplied by this technology is its exploitation of different staining modalities other than H&E, FISH, and IHC, among others. Additionally, PHLI-seq can incorporate machine learning to integrate histopathological information, three-dimensional positions, and genomic alteration landscapes, which will elucidate the relationship between histopathology and genomic and molecular features that have remained obscure based on conventional methods alone. PHLI-seq will make a significant contribution to future subclonal evolutionary analyses of carcinogenesis and the discovery of novel therapeutic targets at the oncogenic subclonal level.

Previously, several groups have developed computational analytic methods for H&E-stained images based on the single nucleus morphology [24, 29], although we utilized image analysis techniques for a different purpose than those of Yuan et al. and Beck et al. We developed a method for image-based grouping of “potential” genetic subclones of cancer cells by grouping cell clusters in an H&E section based on their location and morphology. With the guide of the grouping, we performed PHLI-seq to discover the genetic heterogeneity of the cancer cells. For this purpose, cell cluster-based image analysis would be more suitable than approaches based on single-cell nucleus morphology (see the “Methods” section). However, a limitation of our method is that we did not select the four features based on a large dataset. Yuan et al. and Beck et al. used hundreds of H&E-stained images to establish classifiers based on hundreds of features. As our goal was to develop a technique to reveal subclonal heterogeneity among cancer cells that may have similar cellular phenotype traits, we could not build the image analysis pipeline based on many samples. However, if such a technique can be applied, PHLI-seq would be much more effective. We believe that cutting-edge histopathological image analysis and 3D image visualization techniques can greatly improve the potential and utility of PHLI-seq in the future.

Recently, Navin’s group published a research about multi-clonal invasion in breast tumors using topographic single-cell sequencing, which is an excellent example of spatially resolved sequencing [30]. From a methodological point of view, the research group used an UV-based LCM method and described that UV cutting parameters affected DNA fragmentation and the rate of transfer failure. Also, the research group performed somatic CNA analysis from the dissected single cells, but not SNV analysis which requires much higher quality of amplified DNA in general. Therefore, we expect that the advantage of throughput and IR-based isolation of PHLI-seq will help researchers in studying cutting-edge science in cancer biology by spatially resolved sequencing.

From a clinical perspective, PHLI-seq can be applied to broader areas. As mentioned earlier, genomic regions showing copy number amplifications shared by the three subclones harbor the loci of the oncogenes *MYC*, *ERBB2* (HER2), and *AKT*. In addition, another oncogenic driver mutations are present in subclone 1 (*PIK3CA* and *EPHA3*), subclone 2 (*ATR*), and subclone 3 (*NF1* and *ESR1*). For example, for the subclonal tumorigenic heterogeneity status identified by PHLI-seq in this study, the following insight for a combined therapeutic drug treatment strategy may be proposed. First, to inhibit a background tumor from which the three subclones originated, HER2 inhibitor, MYC inhibitor, and AKT inhibitor might be used. Second, to specifically inhibit subclone 1, PI3K/AKT/mTOR inhibitors and agonistic anti-EPHA3 mAb IIIA4 might be used, given the suppression of the background tumorigenic activity. Third, to prevent subclone 3 specifically, AZD9496 (an oral estrogen receptor inhibitor) might be used. Finally, small molecule ATR inhibitors (ATRi) might be used to specifically prevent subclone 2. As some of the abovementioned drugs are now in nearly final stages of their clinical trials, they may be in use in upcoming years pending FDA approvals. Although such a combined therapeutic strategy could not be applied to patients because our major concern in this study was to develop the novel PHLI-seq approach and the proper timing for its application in patients was lost, we propose that PHLI-seq can be applied to a variety of cancer types in the future to provide insights on subclonal tumorigenic heterogeneity for establishing combined pharmaceutic and treatment strategies, similar to the abovementioned example.

Finally, it should be noted that spatial genetic information can affect clinical interpretations because cancer cells evolve through various geographic conditions and microenvironments in the tissue and can act differently depending on their tumor location. Thus, the PHLI-seq platform meets the needs of cutting-edge cancer biology, which links histopathology to genomics to enable a synergistic and more precise interpretation of cancer.

## Conclusion

In summary, we developed PHLI-seq method which enables high-throughput isolation and genome-wide sequence analysis of a single cell or a small number of cells. PHLI-seq utilizes ITO-based discharging layer and IR laser pulse for isolating cells, and we could analyze both CNA and SNV from the isolated cells. We applied PHLI-seq to an HR-positive/HER2-positive breast tumor and a triple-negative breast tumor for discovering genetic heterogeneity in relation to the 2D or 3D tissue context. For the two cases, we could identify genetic subclones based on subclone-specific CNAs and SNVs. Also, we could identify location, morphology, or phenotype of each genetic subclone.

PHLI-seq could bridge histopathology and cancer genomics by leveraging the map of genomic alteration to the histopathological image of a tissue. We believe that accumulated knowledge in histopathology and advancement of sequencing techniques would converge together through PHLI-seq and offer new insights on the field of cancer research. We envision that PHLI-seq will play a critical role in studying tumor heterogeneity, precision oncology, and cancer genomics.

Although we applied PHLI-seq to genome analysis of tumors, it should be emphasized that PHLI-seq is a general technique for isolating a single cell or a small number cells from a sample on a slide in a high-throughput manner. Therefore, most analysis modality could be accompanied by isolation, including transcriptome and epigenome analysis. We expect that PHLI-seq will serve as an essential tool in a broad range of biological science.

## Methods

### Sample preparation

Human fresh-frozen breast cancer tissues were obtained from the Department of Surgery, Seoul National University Hospital, and analyzed under the approved Institutional Review Board (IRB) protocol (IRB No. 1207-119-420). Frozen breast cancer tissues were stored at − 80 °C until they were sliced into 10-μm-thick sections using a Leica CM3050 cryostat (Leica Microsystems GmbH, Wetzlar, Germany). Tissue sections were thaw-mounted onto ITO-coated glass slides. Glass slides were stored at − 20 °C until analysis. The tissue sections were dried for 15 min at room temperature and subjected to a modified hematoxylin and eosin (H&E) staining technique according to the following protocol: (1) rinse in tap water for 5 min, (2) stain in Harris hematoxylin solution (Merck, Darmstadt, Germany) for 3 min, (3) rinse in tap water (quick dip), (4) rinse in 1% HCl solution/EtOH (quick dip), (5) rinse in tap water for 5 min, (6) counterstain with eosin Y (BBC Biochemical, Mount Vernon, WA) for 3 s, (7) rinse in water (10 dips), (8) dehydrate in 70% EtOH (10 dips), (9) dehydrate in 90% (10 dips), and (10) dehydrate in 100% EtOH (10 dips). Sections were allowed to dry at room temperature.

The tissue used in the 2D experiment was obtained from a 57-year-old woman who underwent total mastectomy with axillary lymph node dissection in April 2014. The breast cancer was a stage IIIC (pT2N3M0, AJCC 7th TNM Staging) IDC which was positive for estrogen receptor (95%), progesterone receptor (2%), and HER-2 (+++/3) on IHC, as evaluated according to the American Society of Clinical Oncology and College of American Pathologists (ASCO/CAP) guidelines. The tissue used for 3D mapping was obtained from a 56-year-old woman who underwent total mastectomy with axillary lymph node dissection in September 2015. The breast cancer was a stage IIA (pT2N0M0, AJCC 7th TNM Staging) IDC which was negative for estrogen receptor (1%), progesterone receptor (negative), and HER-2 (-/3) on IHC.

### Whole-slide imaging to produce digital slides

For whole-slide imaging, a slide was removed from the refrigerator and left at room temperature for 10 min. After placing the slide on an automated microscope (Inverted Microscope Eclipse Ti-E, Nikon Instruments Inc., Melville, NY), we set the scanning region of the slide. Scanning was performed using a × 20 lens, and stitching was carried out by an internal algorithm (NIS-Elements AR Auto Research, Nikon Instruments Inc., Melville, NY).

### Grouping cell clusters based on spatial and phenotypic information

To group the cell clusters based on spatial and phenotypic information, we performed the following steps: (1) whole-slide imaging, (2) cell cluster segmentation, (3) extraction of spatial and phenotypic information, and (4) weighted hierarchical clustering. (1) We imaged the whole slide as described in the “Whole-slide imaging to produce digital slides” section. (2) Cell cluster segmentation was carried out using a conditional random field. The whole-slide image was split into small images of 1024 × 1024 pixels. The learning set was constructed for 50 split images. Then, learning and segmentation were performed using the conditional random field [31]. After segmentation, split images were merged into one image of the original size. (3) We indexed each cell cluster in the merged image using the connected components. Then, four features were extracted for each cell cluster: “position,” “cluster area,” “major axis over minor axis,” and “angle.” Position is the centroid of the cell cluster. Cluster area is the number of pixels of the cell cluster. Major over minor axis is a ratio of the major axis over the minor axis, which estimates how the ellipse-like figure fitted to the cell cluster is. Angle is the angle between the horizontal line and a straight line parallel to a longer side of the rectangle, which estimates the tilt bias of the ellipse-like figure from the vertical line. All scripts were written in C++ and using the OpenCV library for connected components and other features. (4) We performed weighted hierarchical clustering using the four features to find the optimal clustered groups. To find the optimal groups, we maximized a score as follows:

difference between each group/(variance within each group × neighbor ratio of each group),

where

$$ {\displaystyle \begin{array}{c}\mathrm{difference}\ \mathrm{between}\ \mathrm{each}\ \mathrm{group}={\sum}_{n\ne m\in \left\{\mathrm{1..}{N}_c\right\}}{\sum}_{i\in \left\{\mathrm{1..}{N}_f\right\}}\mid {\mu}_{i,m}-{\mu}_{i,n}\mid, \\ {}\mathrm{variance}\ \mathrm{within}\ \mathrm{each}\ \mathrm{group}={\sum}_{m\in \left\{\mathrm{1..}{N}_c\right\}}{\sum}_{i\in \left\{\mathrm{1..}{N}_f\right\}}{\sigma}_{i,m},\\ {}\mathrm{neighbor}\ \mathrm{ratio}\ \mathrm{of}\ \mathrm{each}\ \mathrm{group}={\sum}_{m\in \left\{\mathrm{1..}{N}_c\right\}}{\sum}_{p\in \left\{\mathrm{1..}{N}_m\right\}}\frac{2^{\left(\frac{ND{C}_{m,p}}{NSCm,p}\right)}}{N_m}.\end{array}} $$

Here, *μ*_*i,m*_ and *σ*_*i,m*_ represent the mean and the standard deviation of the *i*^*th*^ feature in the *m*^*th*^ group, *N*_*c*_ and *N*_*f*_ are the number of groups and features, *p* is the index of a cell cluster and *N*_*m*_ is the number of cell clusters in the *m*^*th*^ group. *NSC*_*m,p*_ is the number of cell clusters that are classified into the *m*^*th*^ group among the ten nearest neighboring cell clusters to the *p*^*th*^ cell cluster of the *m*^*th*^ group. In contrast, *NDC*_*m,p*_ is the number of cell clusters that are not classified into the *m*^*th*^ group among the ten nearest neighboring cell clusters to the *p*^*th*^ cell cluster of the *m*^*th*^ group. We maximized the score by fixing the weight for ‘position’ as one, sweeping the weight of the other features from 0.1 to 10 with an increment of 0.25. All scripts were written in R using the stats library for hierarchical clustering (see Additional file 1: Figure S8).

Previously, several groups have developed computational analytic methods of H&E images based on single nuclear morphology [24, 29]. Unlike these approaches, which utilize a single-cell nucleus as a base unit for classifying cell types, we have used the ‘cell cluster’ as the basis for calculations for several reasons. First, we think that the cell types (e.g., cancer, stromal, and immune cells) can be determined based on the single nucleus morphology because their biological functions and phenotypes clearly differ from each other. However, it is hard to predict ‘genetic’ subclones in cancer cells using the single nucleus image because they are all cancer cells and have relatively similar properties (e.g., large and rounded nuclei) in many cases. Therefore, we focused on parameters that can reflect the collective functions of each subclone, such as the growth level, growth direction, and stromal infiltration, which can differ for each subclone. These phenomena are reflected in the morphology of the cell cluster rather than the single nuclei. Second, because a single cell in a tissue section may not contain an intact nucleus, we needed to isolate a cell cluster or a small number of cells in a cell cluster to read the full genomic information. Although we sectioned tissues to a thickness of 10 μm, some cells in the section may have a partial nucleus. We considered it to be a poorly controlled approach to isolate and analyze a single cell in a tissue section. Therefore, we decided to use a cell cluster containing a small number of cells for the sequence analysis. Consequently, we used a cell cluster for image analysis because it was our base unit of sequence analysis. Based on discussions with a pathologist, we selected four features: position, cluster area, major axis over minor axis, and angle. The position and cluster area reflect the relative location of a subclone in a tissue and its growth level, respectively. The major axis over the minor axis is associated with invasiveness and stromal infiltration. Finally, the angle represents the growing direction of a subclone.

### PHLI-seq instrument

The PHLI-seq instrument comprises two motorized stages, a CCD camera, light source, laser source, pulse slit, objective lenses, and fluorescence modules **(**see Additional file 1: Figure S1). The two motorized stages (ACS Motion Control, Migdal, Israel) can be controlled automatically by communicating with a computer. One is for loading sample slides, and the other is for loading tubes to receive isolated cells. The CCD camera (Jenoptik, Jena, Germany) is installed to observe where the laser pulse will be applied through the objective lenses. An Nd:YAG nanosecond laser was purchased from Continuum (Minilite™ Series ML II; Continuum, San Jose, CA). A slit is located in the light path between the laser source and the objective lens to control the region to be isolated. The slit is controlled either manually or automatically to adjust the size of the laser pulse. Objective lenses with various magnifications were purchased from Mitutoyo. The long working distance allows more space between the lens and the sample for user convenience.

### Software to automate the PHLI-seq procedure

We designed two different pieces of software, which were written in Python scripts (see Additional file 8: Supplementary scripts). The source codes are available at Github (https://github.com/BiNEL-SNU/PHLI-seq) and https://zenodo.org with DOI 10.5281/zenodo.1342126. The first was built for the user (a pathologist in our case) to select the cells to be isolated. We shared the whole slide image with the user through a server, and the user ran the software to select the cells of interest while navigating the tissue image through the graphical user interface. After selection, the program produces two files: a text file with locational information about the region of interest, and the image file with the selected targets overlaid with transparent blue on the original image. Both files are required for the automated isolation of the target cells. The second software enables the automatic control of the PHLI-seq instrument. With this software, the users are able to control the slits, change the objective lenses, and move the motorized stages. It also enables automatic target isolation when two files from the first software are loaded. All tissue samples were isolated using an automatic function, while the cell line experiments were performed manually.

### Cell isolation and whole-genome amplification

Cells were isolated from tissue sections, cell lines, or blood smears that were spread on the ITO glass (Fine Chemicals Industry, Seoul, Korea), where ITO was coated on a glass by sputter deposition. An infrared laser was applied to the target area, vaporizing the ITO layer and discharging the target cells in the region. We used glass slides with a 100-nm-thick ITO layer. We tried coatings of 100-nm, 150-nm, and 300-nm ITO layers for the experiments. However, there was no detectable difference between them. Cancer cells could be isolated from 4- to 10-μm-thick tissue sections at all thicknesses. The thickness of the ITO layer can affect the maximum tissue thickness at which the cells can be detached, although we did not undertake a thorough assessment. To separate thicker or harder tissues than in the present study, it may be better to use a thicker ITO layer, although we had no problem separating the cells from the bone tissue when using a 100-nm-thick ITO layer. Using an infrared laser in conjunction with the ITO discharging layer, we could accomplish cell isolation without damaging the cell (see Additional file 1: Figure S9 and Figure S10). The eight-strip PCR tube caps for the retrieval of cells were pre-exposed under O_2_ plasma for 30 s. The cells were lysed using proteinase K (cat no. P4850-1ML, Sigma Aldrich) according to the manufacturer’s directions after the PCR tubes were centrifuged. For whole-genome amplification by multiple displacement amplification, we used GE’s Illustra Genomiphi V2 DNA amplification kit (cat no. 25-6600-30). We added 0.2 μl of SYBR green I (Life Technologies) into the reaction solution for real-time monitoring of the amplification. All amplified products were purified using Beckman Coulter’s Agencourt AMPure XP kit (cat no. A63880) immediately following the amplification reaction. To validate that the samples were thoroughly amplified, we used real-time whole-genome amplification monitoring and PCR validation with in-house designed 16-region primer panels (see Additional file 1: Table S1). To discard poorly amplified samples, we used an MDA product that exhibited an observable amplification level within 40 min after the start of the reaction. Most of the amplified products yielded more than 1 μg, and 800 ng was used for Illumina library construction. To prevent carry-over contamination, the pipette tip, PCR tube, and cap for the reaction were stored in a clean bench equipped with UV light and treated with O_2_ plasma for 30 s before use. Additionally, we monitored the real-time amplification of non-template controls to ensure that no contaminants were transferred.

### Cell isolation by LMD and LPC techniques

We used Leica LMD6500 (Leica Microsystems, Wetzlar, Germany) to perform LMD and LPC for cell isolation (see Additional file 4: Video S3, Additional file 5: Video S4). The experiment was supported by an expert from the supplier of the instrument. We used nuclease and human nucleic acid-free PET-membrane FrameSlide (Leica Microsystems) to prepare samples. To minimize UV damage to cells, laser power was adjusted down to a minimum level (40~45 in the operating software for LMD6500). For LPC, the aperture in the instrument was opened to maximum and the laser was out-focused. Other experimental procedures were same with PHLI-seq.

### Sequencing of the amplified genome

The whole-genome-amplified products or genomic DNA extracts were fragmented using an EpiSonic Multi-Functional Bioprocessor 1100 (Epigentek) to generate a 150~250-bp fragment distribution. The fragmented products underwent Illumina library preparation for end repair, 3′dA-tailing, adaptor ligation, and PCR amplification according to the manufacturers’ instructions. We used the Celemics NGS Library Preparation Kit (LI1096, Celemics, Seoul, Korea) for the whole-genome sequencing library preparation, SureSelectXT (Agilent, CA, USA) for whole-exome sequencing, and the Celemics Customized Target Enrichment Kit (SICT96, Celemics, Seoul, Korea) for targeted sequencing. DNA purification was performed by ^TOPQ^XSEP MagBead (XB6050, Celemics, Seoul, Korea), and DNA libraries were amplified using the KAPA Library Amplification Kit (KAPA Biosystems, KK2602). Finally, the products were quantified by TapeStation 2200 (Agilent, CA, USA). We used a HiSeq 2500 50SE (Illumina) to generate 0.16 Gb/sample for whole-genome sequencing and a HiSeq 2500 150PE (Illumina) to generate 5 G/sample and 0.88G/sample for whole-exome and targeted sequencing, respectively.

### Targeted sequencing panel

We chose 121 genes for the SNUH BCC (Seoul National University Hospital Breast Care Center Panel) based on the following criteria.

In our previous study, we performed whole-exome sequencing and RNA-Seq of 200 pairs of matched clinical breast cancer and normal samples from Korean breast cancer patients. In addition, we analyzed the mutations, CNAs, and gene expression results of approximately 3000 clinical breast cancer samples in the TCGA and METABRIC databases. Based on these study results, we chose the genes that showed a high frequency and recurrent mutations, genomic copy number amplifications and deletions, and expression changes in breast cancer samples, which could be oncogenes, tumor suppressor genes, or breast cancer-associated genes. Among the 121 genes that we chose based on such criteria were previously known oncogenes, tumor suppressor genes, and breast cancer-associated genes, including genes involved in DNA repair pathways.

However, our SNUH BCC panel is unique compared with other cancer panels based on NGS because it includes a certain portion of novel breast cancer-associated genes that have not been included in other recent popular and conventional cancer panels. In this regard, our SNUH BCC panel is not only targeted to worldwide breast cancer patients but is also ethnically directed to Korean breast cancer patients for diagnosis and therapeutic prognostic prediction.

### Sequence alignment and preprocessing

The NGS sequence reads were mapped to the GRCh37 human reference genome using BWA-MEM [32] (version 0.7.8) with default parameters. The resulting SAM files are sorted by chromosome coordinates, followed by PCR duplication marking using Picard (version 1.115) (http://broadinstitute.github.io/picard/). Reads with a mapping quality score less than 30 or that have a supplementary alignment were removed from the BAM file before the subsequent analysis.

### Detecting copy number alterations

We used low-depth whole-genome sequencing data and the variable-size binning method [33] to estimate the CNAs of the samples. Briefly, the whole genome was divided into 10,000 variable-sized bins (median genomic length of bin = 276 kbp) in the case of breast cancer tissue samples and 7,000 bins (median = 396 kbp) in the case of cell line samples, in which each bin had an equal expected number of uniquely mapped reads. Then, each NGS sequence read was assigned to each bin followed by Lowess GC normalization to obtain the read depth of each bin. The copy number was estimated by normalizing the read depth of each bin by the median read depth of the reference DNA.

### Clustering samples based on the CNA dataset

After the CNA detection followed by MergeLevels [34], the data underwent multi-sample segmentation with gamma = 20. Given the multi-sample segmentation, the event vector was constructed for each cell by providing a value of 0 for the segments with a median copy number of reference DNA, 1 for segments with a gain, and − 1 for segments with a loss. Finally, a correlation matrix was constructed, and hierarchical clustering was performed. To evaluate the accuracy of the clustering, a multiscale bootstrapping resampling method was used to approximately calculate the unbiased *p* value [35]. The approximately unbiased (AU) *p* value indicated the strength of the cluster supported by bootstrapping. Bootstrap samples were generated, and clustering analysis was applied to them repeatedly. The AU *p* value of a cluster was related to the frequency that appeared in the bootstrap replicates, and the method was implemented by the R package Pvclust [36].

### Single nucleotide variant detection

Before SNV detection, GATK (v3.5-0) IndelRealigner and BaseRecalibrator were used to locally realign reads around the Indel and recalibrate the base quality score of BAM files [37]. We then used three different variant callers (GATK UnifedGenotyper, Varscan, and MuTect) and combined the results to avoid false-positive variant detection [38]. First, GATK UnifiedGenotyper was used with default parameters followed by GATK VariantRecalibrator to obtain filtered variants [37]. Tumor bulk sample and PHLI-seq sorted sample data were processed together to produce a single vcf file. The training data used for variant recalibration included dbSNP build 137, hapmap 3.3, Omni 2.5, and 1000G phase1, and QD, MQ, FS, ReadPosRankSum, and MQRankSum annotations were used for the training. Variants detected in the paired blood sample of the cancer patient were removed to produce the final list of GATK called variants. Varscan2 [39] (ver 2.3.7) and Mutect [40] (ver 1.1.4) were used with default parameters to produce the lists of Varscan and MuTect called variants, respectively. Here, paired blood read data was also used to separately call germline mutations.

Among the variants detected in the samples by the three variant callers, variants called by at least two callers were collected to obtain intra-sample double called sites. By considering only these variants for subsequent analysis, we could reduce false-positive variant detection derived from NGS errors [38]. Among the intra-sample double called sites, variants found in at least two samples were collected to remove WGA (whole-genome amplification) errors, and the genomic loci with the resultant variants were considered confident sites. Finally, a variant in the confident sites was considered to be true if one of the three variant callers detected the variant at the locus and the allele count of the variant was significantly larger than that of the other non-reference bases (Fisher’s exact test, *p* < 10e−4).

### Single-molecule deep sequencing to validate detected SNVs

We carried out single-molecule deep sequencing [41] to validate the SNVs detected from the tumor bulk genomic DNA extract. We randomly selected a portion of mutations detected by PHLI-seq and generated a sequencing library for the targeted sites by tagging unique molecular barcodes to each DNA molecule. The difference from the referred paper is that we generated a targeted library using PCR with minimal cycles before tagging the molecular barcodes. This would lose the ability to call duplex consensus sequences (DCSs), but it is still possible to call single-strand consensus sequences (SSCSs). We performed 3-plex PCR using 50 ng of genomic DNA with a 2X KAPA2G Fast Multiplex kit (KAPA Biosystems, KK5802). The amplified products underwent gel electrophoresis, gel purification, and quantification by a Qubit high-sensitivity dsDNA quantification kit (Invitrogen), and normalization for pooling. The pooled library was end-repaired and dA-tailed (NEB, NEBNext® End Repair Module, E6050S). Then, the duplex tags were ligated, and DNA molecules with a length of 200 to 1000 bp were purified to remove dimers. Finally, the molar concentration of the tag-ligated libraries was quantified (see Additional file 1: Figure S11). The constructed library was sequenced by MiSeq 250 PE (Illumina) to generate a 18,182X (median) single-molecule sequencing depth. After generating the SSCSs, the targeted loci (where SNV had been detected) and background loci (where no SNV had been detected) were split. Given the allele frequency distribution from the background group, we prepared the true positive call set from the targeted loci (Benjamini–Hochberg false discovery rate < 0.05).

### Defining cancer genes

After the CNA and SNV detection, potential driver alterations were annotated for cancer-related genes. The gene list consists of 682 genes, which were compiled from The Cancer Gene Census [42] and The Cancer Gene Atlas (TCGA) Project [27, 43].

## Additional files

Additional file 1 Supplementary notes, supplementary figures, and supplementary tables. (DOCX 19232 kb)

Additional file 8 Supplementary scripts. (ZIP 15961 kb)

## Abbreviations

ADH: Atypical ductal hyperplasia
ADO: Allele dropout
CNA: Copy number alteration
DCIS: Ductal carcinoma in situ
FISH: Fluorescence in situ hybridization
FPR: False-positive rate
H&E: Hematoxylin and eosin
HER2: Human epidermal growth factor receptor 2
HR: Hormone receptor
IDC: Invasive ductal carcinoma
IHC: Immunohistochemistry
IR: Infrared
ITO: Indium tin oxide
MDA: Multiple displacement amplification
PHLI-seq: Phenotype-based high-throughput laser-aided isolation and sequencing
SNV: Single nucleotide variant
UV: Ultraviolet
WES: Whole-exome sequencing

## References

[1] Goodwin S, McPherson JD, McCombie WR (2016). Coming of age: ten years of next-generation sequencing technologies. Nat Rev Genet.

[2] Burrell Rebecca A., McGranahan Nicholas, Bartek Jiri, Swanton Charles (2013). The causes and consequences of genetic heterogeneity in cancer evolution. Nature.

[3] Roth A (2014). PyClone: statistical inference of clonal population structure in cancer. Nat Methods.

[4] Jiao W, Vembu S, Deshwar AG, Stein L, Morris Q (2014). Inferring clonal evolution of tumors from single nucleotide somatic mutations. BMC Bioinf.

[5] Carter SL (2012). Absolute quantification of somatic DNA alterations in human cancer. Nat Biotechnol.

[6] Gerlinger M, Rowan AJ, Horswell S, Larkin J (2012). Intratumor heterogeneity and branched evolution revealed by multiregion sequencing. N Engl J Med.

[7] Yates LR (2015). Subclonal diversification of primary breast cancer revealed by multiregion sequencing. Nat Med.

[8] Lindberg J (2013). Exome sequencing of prostate cancer supports the hypothesis of independent tumour origins. Eur Urol.

[9] Casasent AK, Edgerton M, Navin NE (2017). Genome evolution in ductal carcinoma in situ: invasion of the clones. J Pathol.

[10] Espina V (2006). Laser-capture microdissection. Nat Protoc.

[11] Vandewoestyne M, Goossens K, Burvenich C, Van Soom A, Peelman L, Deforce D (2013). Laser capture microdissection: should an ultraviolet or infrared laser be used?. Anal Biochem.

[12] Wang Y (2014). Clonal evolution in breast cancer revealed by single nucleus genome sequencing. Nature.

[13] Navin NE (2015). The first five years of single-cell cancer genomics and beyond. Genome Res.

[14] Wang Y, Navin NE (2015). Advances and applications of single-cell sequencing technologies. Mol Cell.

[15] Zafar H, Wang Y, Nakhleh L, Navin N, Chen K (2016). Monovar: single-nucleotide variant detection in single cells. Nat Methods.

[16] Gao R (2016). Punctuated copy number evolution and clonal stasis in triple-negative breast cancer. Nat Genet.

[17] Sinn HP, Kreipe H (2013). A brief overview of the WHO classification of breast tumors, 4th edition, focusing on issues and updates from the 3rd edition. Breast Care.

[18] Markowetz F (2016). A saltationist theory of cancer evolution. Nat Genet.

[19] Ke R (2013). In situ sequencing for RNA analysis in preserved tissue and cells. Nat Methods.

[20] Junker JP (2014). Genome-wide RNA tomography in the zebrafish embryo. Cell.

[21] Achim K (2015). High-throughput spatial mapping of single-cell RNA-seq data to tissue of origin. Nat Biotechnol.

[22] Satija R, Farrell J a, Gennert D, Schier AF, Regev A (2015). Spatial reconstruction of single-cell gene expression data. Nat Biotechnol.

[23] Kothari S, Phan JH, Stokes TH, Wang MD (2013). Pathology imaging informatics for quantitative analysis of whole-slide images. J Am Med Inform Assoc.

[24] Yuan Y., Failmezger H., Rueda O. M., Ali H. R., Graf S., Chin S.-F., Schwarz R. F., Curtis C., Dunning M. J., Bardwell H., Johnson N., Doyle S., Turashvili G., Provenzano E., Aparicio S., Caldas C., Markowetz F. (2012). Quantitative Image Analysis of Cellular Heterogeneity in Breast Tumors Complements Genomic Profiling. Science Translational Medicine.

[25] Schütze K, Lahr G (1998). Identification of expressed genes by laser-mediated manipulation of single cells. Nat Biotechnol.

[26] The Cancer Genome Atlas Network (2012). Comprehensive molecular portraits of human breast tumours. Nature.

[27] Kandoth C (2013). Mutational landscape and significance across 12 major cancer types. Nature.

[28] Navin N (2011). Tumour evolution inferred by single-cell sequencing. Nature.

[29] Beck AH (2011). Systematic analysis of breast cancer morphology uncovers stromal features associated with survival. Sci Transl Med.

[30] Casasent AK (2018). Multiclonal invasion in breast tumors identified by topographic single cell sequencing. Cell.

[31] Domke J (2013). Learning graphical model parameters with approximate marginal inference. IEEE Trans Pattern Anal Mach Intell.

[32] Li H, Durbin R (2009). Fast and accurate short read alignment with Burrows-Wheeler transform. Bioinformatics.

[33] Baslan T (2012). Genome-wide copy number analysis of single cells. Nat Protoc.

[34] Willenbrock H, Fridlyand J (2005). A comparison study: applying segmentation to array CGH data for downstream analyses. Bioinformatics.

[35] Shimodaira H (2004). Approximately unbiased tests of regions using multistep-multiscale bootstrap resampling. Ann Stat.

[36] Suzuki R, Shimodaira H (2006). Pvclust: an R package for assessing the uncertainty in hierarchical clustering. Bioinformatics.

[37] McKenna A (2010). The Genome Analysis Toolkit: a MapReduce framework for analyzing next-generation DNA sequencing data. Genome Res.

[38] Lodato MA (2015). Somatic mutation in single human neurons tracks developmental and transcriptional history. Science (80-. ).

[39] Koboldt DC (2012). VarScan 2: somatic mutation and copy number alteration discovery in cancer by exome sequencing. Genome Res.

[40] Cibulskis K (2013). Sensitive detection of somatic point mutations in impure and heterogeneous cancer samples. Nat Biotechnol.

[41] Kennedy SR (2014). Detecting ultralow-frequency mutations by Duplex Sequencing. Nat Protoc.

[42] Forbes SA (2015). COSMIC: exploring the world’s knowledge of somatic mutations in human cancer. Nucleic Acids Res.

[43] Ciriello G (2015). Comprehensive molecular portraits of invasive lobular breast cancer. Cell.

[44] Kim S, et al. Performance comparison between PHLI-seq, LMD, and LPC: BioProject; 2018. https://www.ncbi.nlm.nih.gov/bioproject/PRJNA485411

[45] Kim S, et al. Collection and sequencing efficiencies of PHLI-seq: BioProject; 2018. https://www.ncbi.nlm.nih.gov/bioproject/PRJNA485407

[46] Kim S, et al. Test for laser induced damage of PHLI-seq: BioProject; 2018. https://www.ncbi.nlm.nih.gov/bioproject/PRJNA485405

[47] Kim S, et al. PHLI-seq analysis of a HER2-positive breast cancer tissue: BioProject; 2018. https://www.ncbi.nlm.nih.gov/bioproject/PRJNA485402

[48] Kim S, et al. Duplex sequencing validation of PHLI-seq: BioProject; 2018. https://www.ncbi.nlm.nih.gov/bioproject/PRJNA485418

[49] Kim S, et al. PHLI-seq analysis of a TNBC tissue: BioProject; 2018. https://www.ncbi.nlm.nih.gov/bioproject/PRJNA485420

[50] Kim S, Lee AC, Kim J, Kwon S. PHLI-seq: Zenodo; 2017. 10.5281/zenodo.1342126

