# Supplementary material for: PHLI-seq: constructing and visualizing cancer genomic maps in 3D by phenotype-based high-throughput laser-aided isolation and sequencing
Source: Genome Biol. 2018 Oct 8;19:158. doi: 10.1186/s13059-018-1543-9 (PMC6176506; doi:10.1186/s13059-018-1543-9)
Supplement: Supplementary file 7 — Supplementary notes, supplementary figures, and supplementary tables. (DOCX 19232 kb) [file 13059_2018_1543_MOESM1_ESM.docx]

**Supplementary Information[**

# **Note S1.** For isolating cells using PHLI-seq instrument, we used a retrieving cap holder described by the figures below. This is a custom device to hold twelve 8-strip caps, which enable to capture up to 96 samples in a single run. We designed the retrieving cap holder for our convenience, and it’s not a prerequisite. The retrieving cap holder comprises two parts. One is a rack structure where twelve 8-strip caps can be mounted. The other is beam structures, which press down the 8-strip caps. The beam structures are attracted toward the rack structure by magnetic force, by which the 8-strip caps are held tightly.


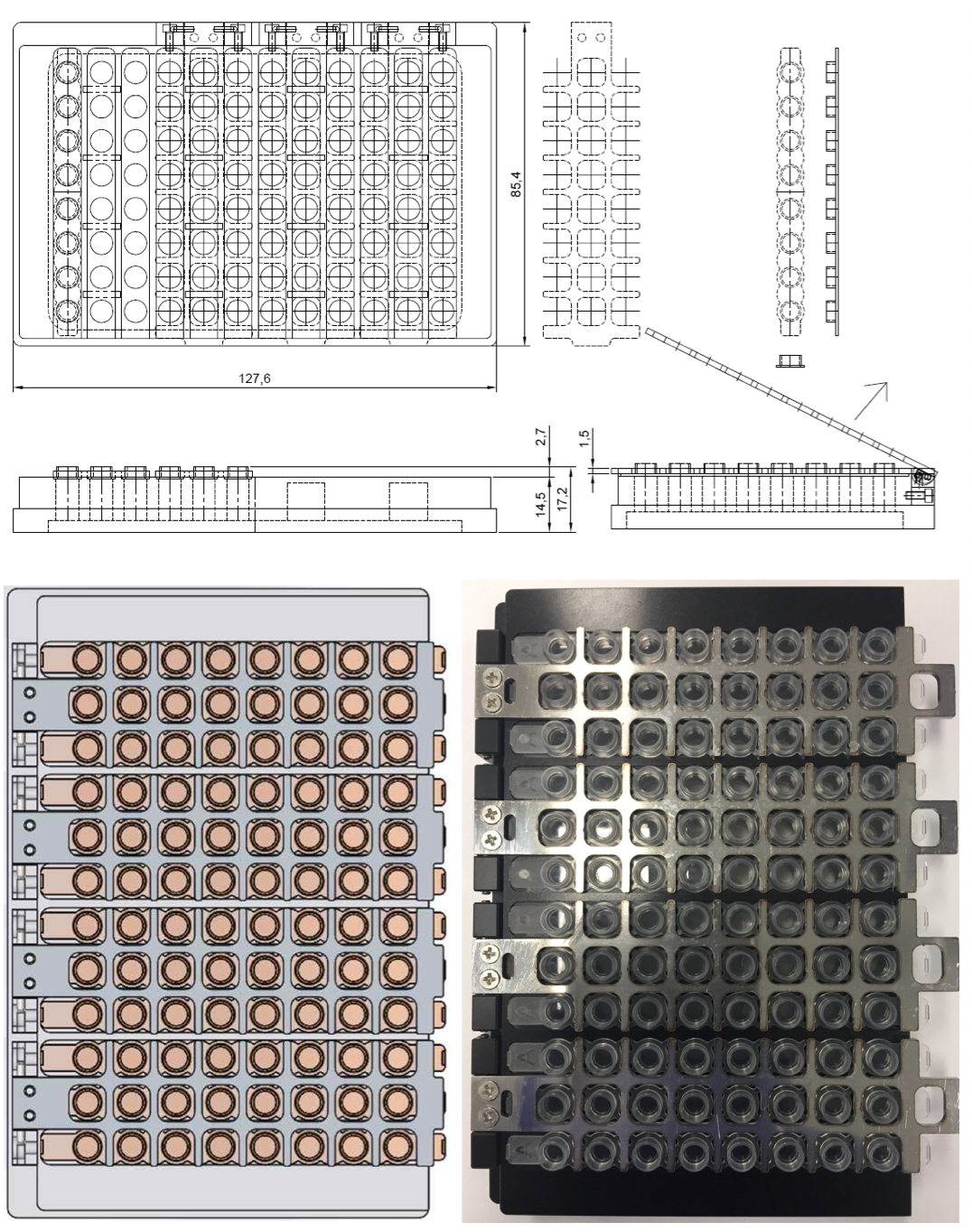


The figure below shows the situation where the above retrieving cap holder is mounted on the PHLI-seq equipment to isolate cells. The thickness of the slide glass holder is about 1.3 mm, and sample-to-cap distance is about 1.4~1.5 mm in actual operation. The retrieving cap has an inner diameter of 4.5 mm. Therefore, when the cell is separated at an angle of under 60 degrees, it is accurately inserted into the cap. The separation angle for not entering the other cap is about 72 degrees.


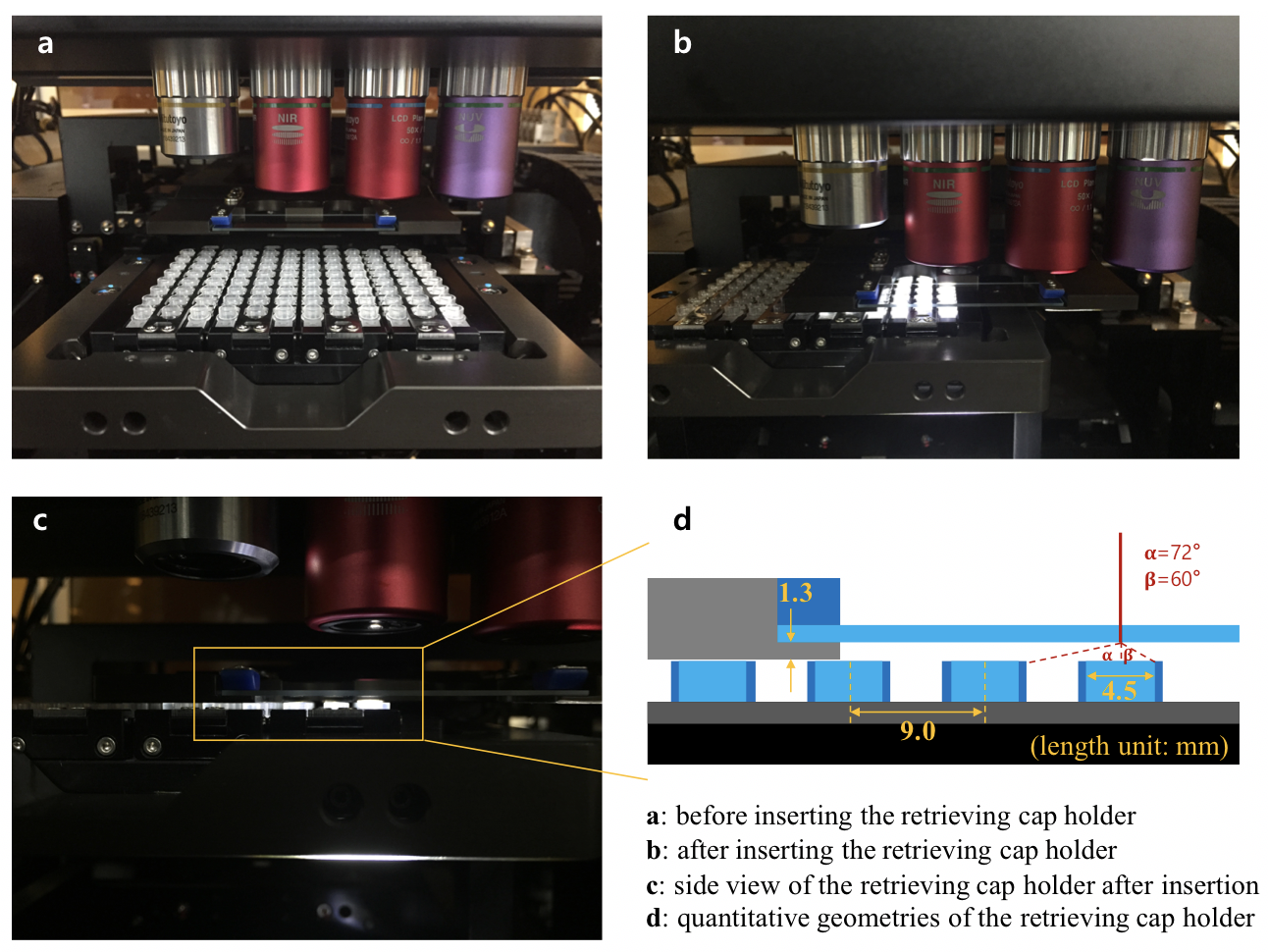


**Note S2.** PHLI-seq performance validation

To characterize and validate the quality of PHLI-seq according to whole genome amplification methods and the number of input cells, single or twenty cells’ whole genomes of a cell line were amplified by multiple displacement amplification (MDA) or multiple annealing and looping based amplification cycles (MALBAC). First, we evaluated coverage breadth and uniformity of each group and found that coverage breadth in twenty-cell group (88.18%± 0.70%) was almost equivalent to that of the bulk population (90.92%) (Fig. S2). In contrast, single-cell group showed limited coverage (77.8%±3.30%) compared to twenty-cell group (P=0.0079, Wilcoxon rank sum test). Though there was significant difference of coverage breadth and uniformity between single- and twenty-cell groups, there was no difference between MDA and MALBAC.

In respect of allele dropout (ADO), single-cell group showed 18% to 40% of dropout rate, and MDA showed lower ADO rate than MALBAC. In contrast, much lower ADO rates were observed in twenty-cell group (P = 0.0285, Wilcoxon rank sum test), showing 0.956% on average, which is 32.3 fold reduced dropout rate compared to the single-cell group (Fig. S3). On the other hand, false positive rate (FPR) in single-cell and twenty-cell groups, which were amplified by MDA were 2.13 × 10-5 and 5.65 × 10-6 respectively. However, MALBAC showed much higher FPR in both groups (8.4 × 10-4 and 5.1 × 10-5 from single-cell and 20-cells group, respectively) (Fig. S3). This could be attributed to BstI polymerase used in MALBAC.

Moreover, we found that only reaction from twenty cells amplified by MDA had similar allele frequency distribution with that of gDNA from population of cells (Fig. S3). The distribution from population of cells was normally distributed, but amplified products presented bimodal or skewed distribution. This result could be ascribed to amplification bias which makes detected allele frequency of a locus deviate from the original value, and polymerase error which creates a new population in a low range of allele frequency. Bringing results altogether, we concluded that amplifying about twenty cells by MDA is appropriate for reducing effects of amplification bias and polymerase error.

**Note S3. Applying PHLI-seq to hormone receptor-positive/Human epidermal growth factor receptor 2-positive breast cancer**

A tissue section of a preserved fresh frozen sample was prepared and stained with hematoxylin & eosin (H&E). The prepared section was scanned using an automated microscope to generate a high-resolution whole-section image (see “Methods” and Additional file 1: Fig. S4a). The image was segmented into cell clusters to generate a binary image, and spatial information and morphological phenotypes were extracted from each cell cluster. Then, a grouping of the cell clusters was performed using weighted hierarchical clustering to generate six groups (see “Methods” and Additional file 1: Fig. S8 and Fig. S4b). Based on an additional inspection by pathologists, 53 cell clusters were selected from the groups for analysis by PHLI-seq (see Additional file 1: Fig. S4c and d). With an average of 20-30 cells in each cell cluster, whole-genome amplification and quality filtering were performed (see “Methods”).

First, genome-wide CNA analysis by low-depth whole-genome sequencing revealed three major subclonal populations in the tumor sample (**Fig. 4a** and **b**, approximate unbiased p-value > 0.99, multiscale bootstrap resampling with 10,000 iterations, see “Methods”). The three subclonal populations had both shared and unique alteration profiles. The shared alterations include 1q gain, 8q gain, 8p loss and HER2 amplifications, all of which had been previously reported as frequent CNAs in human breast cancer and other types of cancer [1, 2]. In this sample, the causative fitness gains underlying breast carcinogenesis appeared to take place in the *MYC* (8q24), *AKT3* (1q44), or *ERBB2* (HER2) (17q12) regions, all of which were gained or amplified in 12.4%~21.3% of cases in The Cancer Genome Atlas (TCGA) project. Additionally, the up-regulation of these genes has been known to be associated with the causation of breast cancer [1]. In contrast, subclones 1, 2 and 3 harbor unique gains or focal amplifications at 6q22.32-q23.2, 2q14.1-q14.2 and 8p21.3-p22, respectively. Among them, the amplification of *CUG2* (Cancer-Upregulated Gene 2, also called *CENPW*, 6q22.32), which is known to be associated with tumor progression [3], might contribute to the high proliferative ability observed in subclone 1.

To investigate somatic SNV, we performed targeted sequencing of 121 genes associated with breast cancer (see “Methods” and Additional file 1: Table S2). The results revealed unique mutational profiles in each subclone, consistent with those determined by whole-genome sequencing (**Fig. 4c**). In our targeted sequencing analysis of 53 cell cluster samples, we found that mutations in *PIK3CA*, *EPHA3*, *KIT*, *ERBB4* and *KMT2C* occurred in subclone 1, mutations in *KMT2C*, *ATR* and *KDM5B* in subclone 2, and mutations in *TOP2A*, *NF1*, *ESR1*, *JAK1* and *MST1R* in subclone 3. Notably, PIK3CA p.M1043I, which has previously been known to be an oncogenic mutation that causes an increase in PI3K lipid kinase activity, constitutive AKT activation and the transformation of NIH3T3 cells and chick embryo fibroblasts, was shared between subclones 1 and 3 [4, 5]. In addition, PIK3CA p.M1043I and EPHA3 p.E794K in subclone 1 and ATR p.R2363X in subclone 2 have been reported to be recurrently observed in breast and other types of cancer [2, 6]. Intriguingly, the stop-gain mutation ATR p.R2363X destroyed the PI3_PI4_kinase domain, a key functional structure, in the tumor suppressor ATR, which plays a role in the DNA mismatch repair pathway. Another validated mutation that could cooperate in driving the subclonal heterogeneity is ESR1 p.E380Q in subclone 3, which has been known to be associated with endocrine therapy resistance in a breast cancer xenograft model [7]. Although the nonsynonymous EPHA3 p.E794K is a novel mutation that has not been reported to date in dbSNP, we confirmed that this mutation with the highest deleterious risk scores (SIFT_score, 0 and Polyphen2_HDIV_score, 1) occurred in the protein tyrosine kinase domain (PF07714) of EPHA3, meriting further functional analysis in the future. The stop-gain mutation KMT2C p.Q3417X in subclone 1 completely truncated the SET domain (PF00856), a key functional structure, implying a consistency with a recent report showing that low KMT2C expression is associated with a poor outcome in ER-positive breast cancer patients [8]. In cooperation with the oncogenic copy number amplifications mentioned above, those deleterious mutations in the three subclones may contribute significantly to the independent oncogenic evolution of each subclone.

For further analysis, we performed whole-exome sequencing of four samples selected from each subclone (**Fig. 4d**). The mutations detected in the tumor bulk mainly covered the mutational profile in subclone 1 (78.8% of exclusive mutations), whereas only approximately 6.81% of the mutations in subclone 3 were detected in the tumor bulk (see Additional file 1: Fig. S13). This result implies that PHLI-seq can provide rich information about subclonality and variants with a low-level allele fraction in heterogeneous tumors, even those with subclones that are too minor to be detected by conventional methods.

Next, we validated our SNV analysis by single-molecule deep sequencing. We randomly selected a portion of mutations that were detected using PHLI-seq and generated a sequencing library for the targeted sites by tagging a unique molecular barcode to each DNA molecule to precisely discriminate the next-generation sequencing results from errors (see “Methods” and Additional file 1: Fig. S11). Sequencing was performed to read the targeted region with an 18,182-fold single-molecule read depth. We set our validation limit to 0.55% to limit the false discovery rate to < 0.05. The results validated 92.3% (12/13) of the subclonal mutations detected by targeted sequencing (see Additional file 1: Fig. S14). Moreover, 72.2% (78/108) of the mutations observed in the whole-exome sequencing were validated. The validation rate was 90.9% (50/55) when mutations that were observed in more than three samples were considered (see Additional file 1: Fig. S14).

Based on the CNA and SNV analyzes, we could infer the evolutionary history of the subclones in the tumor (see Additional file 1: Fig. S6). An ancestral precursor clone could be generated by gaining an advantage for tumorigenic proliferation by accumulating the CNAs and mutations shared by the major three subclones. However, we cannot rule out the possibility that the genesis of CNAs and mutations shared by the three subclones might have been completed over several generations of previous initial subclones originating from a *bona fide* ancestor clone, giving rise to the birth of a plausible precursor clone that harbored the variants shared by the three subclones. Then, one cell in the initial precursor clone could have acquired other variants, becoming a subsequent precursor clone for the generation of subclones 1 and 3. Other cells in the initial precursor clone could accumulate different variants that could consequently give rise to subclone 2, evolutionarily branching out from subclones 1 and 3. This result can be inferred from the observation that subclones 1 and 3 have many common mutations, including PIK3CA p.M1043I, whereas subclone 2 has exclusive mutations and mutations that are common to all three subclones. Moreover, a copy number analysis of the *HER2* gene showed that subclone 2 has a much higher *HER2* amplification level than subclones 1 and 3, further supporting this scenario (see Additional file 1: Fig. S4e and f). After being split into the two lineages, the precursor subclones would have accumulated copy number changes or mutations independently, and one of the two could be further split to ultimately give rise to subclones 1 and 3. Subclone 3 could have accumulated more mutations, potentially because of the exclusive mutation in the MSH2 gene encoding a component of the post-replicative DNA mismatch repair system.

**Figure S1.** PHLI-seq instrument is comprised of two motorized stages, CCD camera, light source, laser source, laser shape modulating slit, objective lenses, and fluorescence modules. The entire system is controlled by in-house control software.


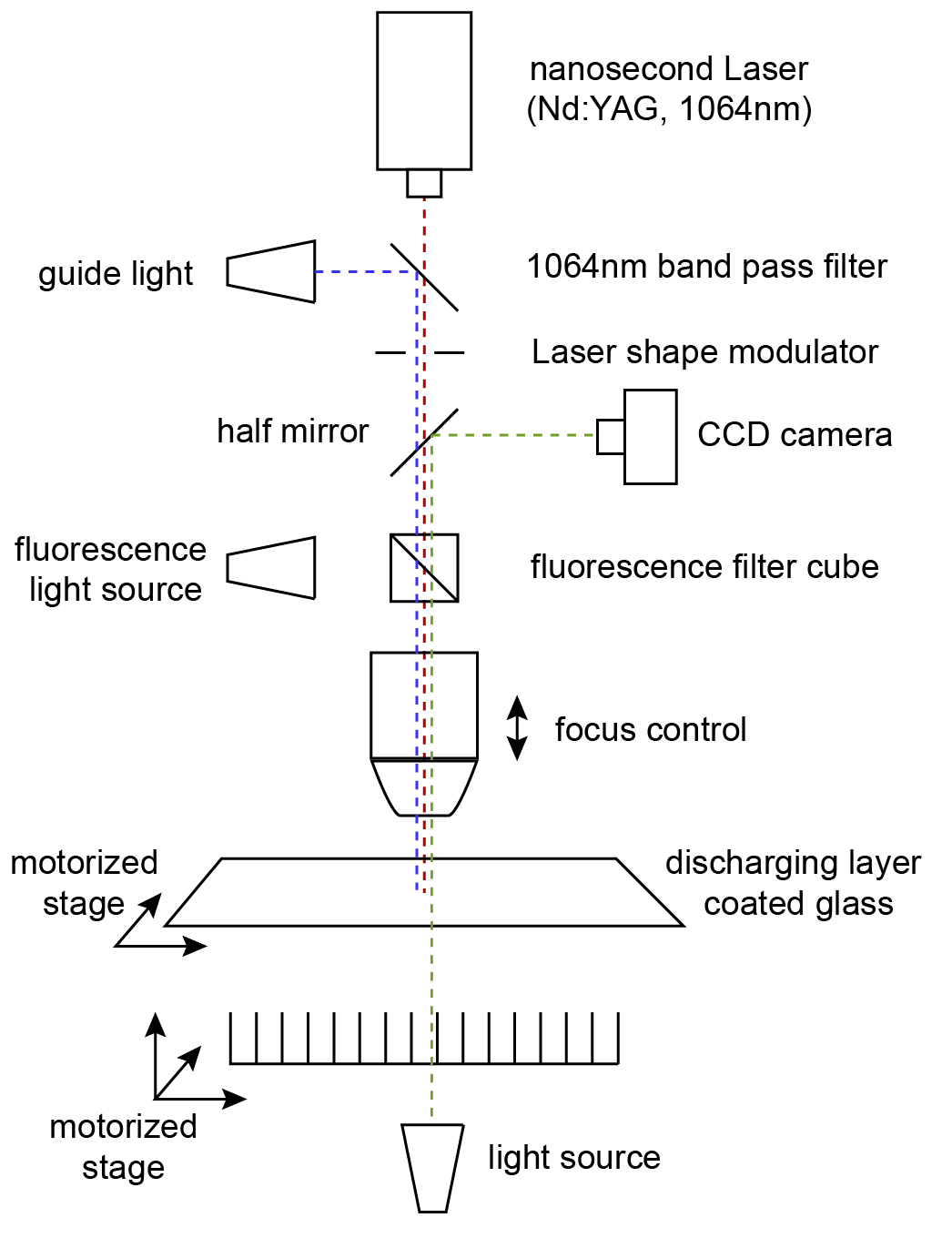

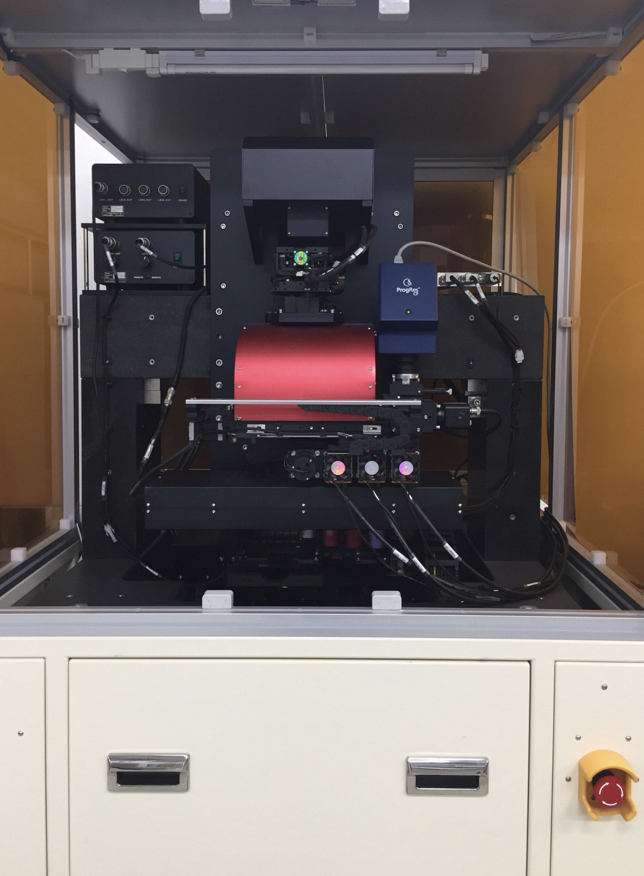


**Figure S2.** Sequencing quality validation of PHLI-seq. HL60 cells were isolated by PHLI-seq and whole genome amplified by MDA or MALBAC. Amplified DNA underwent low depth whole genome sequencing: (a) Bin coverage breadth according to sequencing depth and preparation methods. Dotted lines represent 20-cell isolation, and solid line is for single cell isolation. Sample ‘Dilution’ indicates sequencing result of single cells isolated by critical dilution, not PHLI-seq, and amplified by MDA; (b-c) Copy number plot according to genome position.


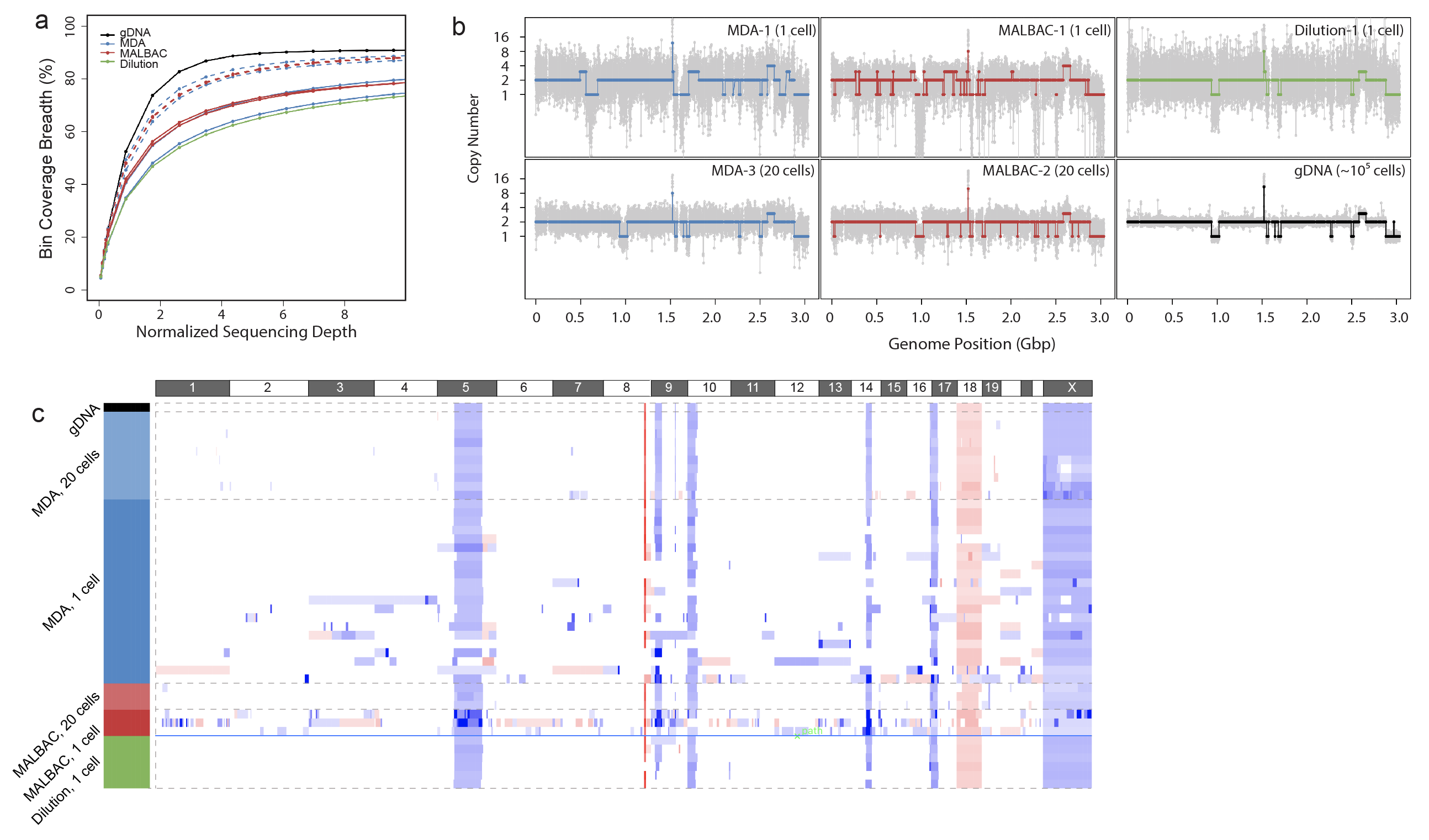


**Figure S3.** Sequencing quality validation of PHLI-seq. HL60 cells were isolated by PHLI-seq and whole genome amplified by MDA or MALBAC. Amplified DNA was analyzed whole exome sequencing (WES) to calculate allele dropout (ADO) and false positive rate (FPR): (a) Calculated ADO and FPR; (b) Allele frequency distribution according to preparation methods.


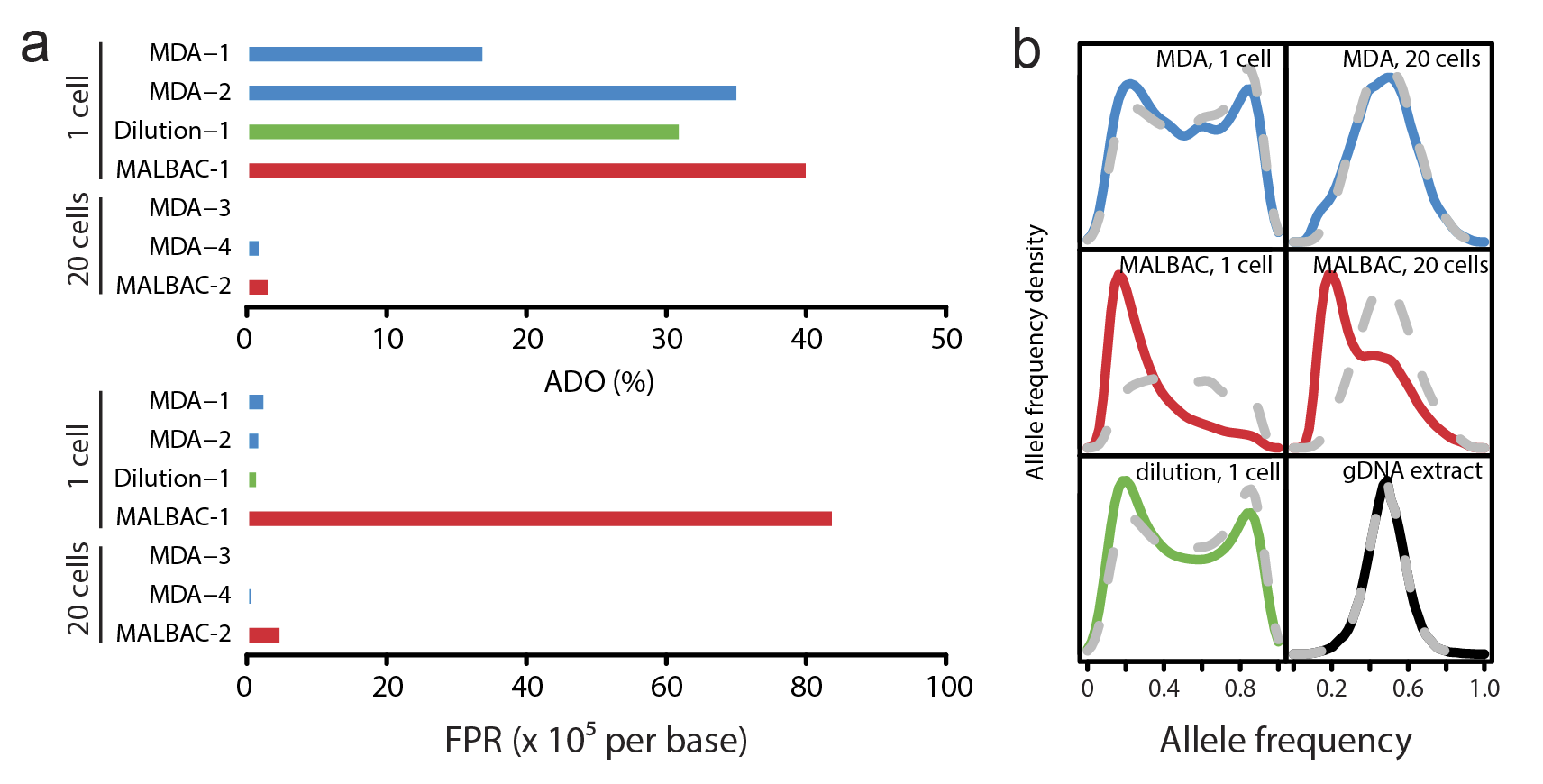


**Figure S4.** Fifty-three cell clusters were isolated and sequenced by PHLI-seq after grouping the cell clusters in HER2-positive breast cancer based on their location and morphology. (a) The H&E tissue section was imaged using a whole-slide imaging system. The enlarged image shows a typical example of a cell cluster. We highlighted the boundary of the cell cluster with a white solid line. (b) The H&E section image was segmented into cell clusters to generate the binary image. The cell cluster locations and three morphological features were extracted for grouping. Based on the information, the cell clusters were grouped by a weighted hierarchical clustering method to generate six groups, which are represented by six distinct colors. (c) Tissue section imaged after cell isolation. (d) Tissue images before and after isolation by the PHLI-seq system. The shape of the infra-red laser pulse can be modulated to isolate various shapes of cell clusters. (e and f) HER2 gene copy numbers in subclones identified by whole genome sequencing analysis and their respective FISH images.


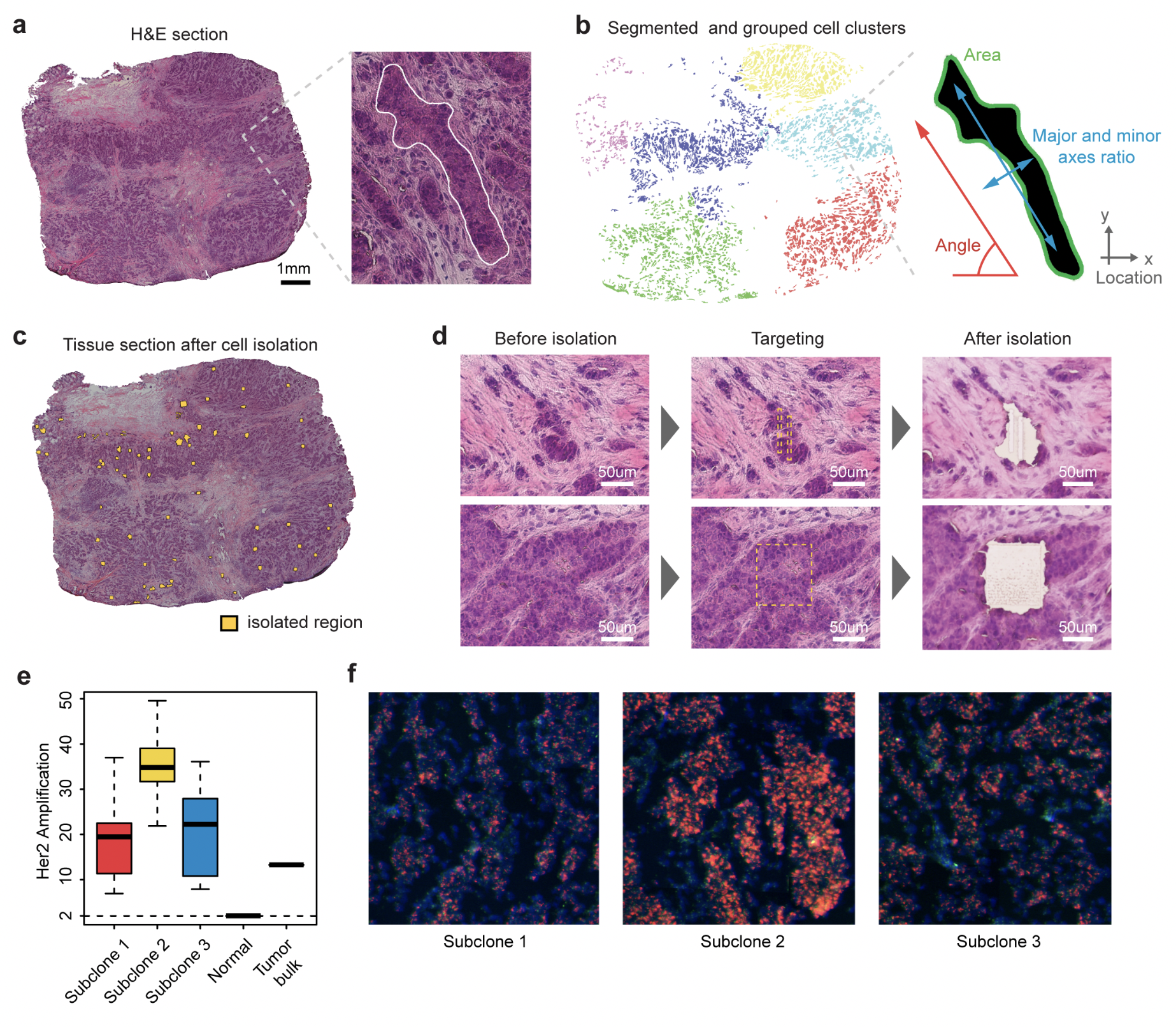


**Figure S5.** We isolated additional cell clusters (n=27) at the boundaries between subclones. The isolated samples were analyzed by low-depth whole-genome sequencing. Then, clustering analysis was performed based on the inferred copy number data for both previously isolated (n=53) and additionally isolated samples (n=27). The results showed that the 80 cell clusters from the HER2-positive tissue sections were classified into one of the three previously defined cancer subclones.

**
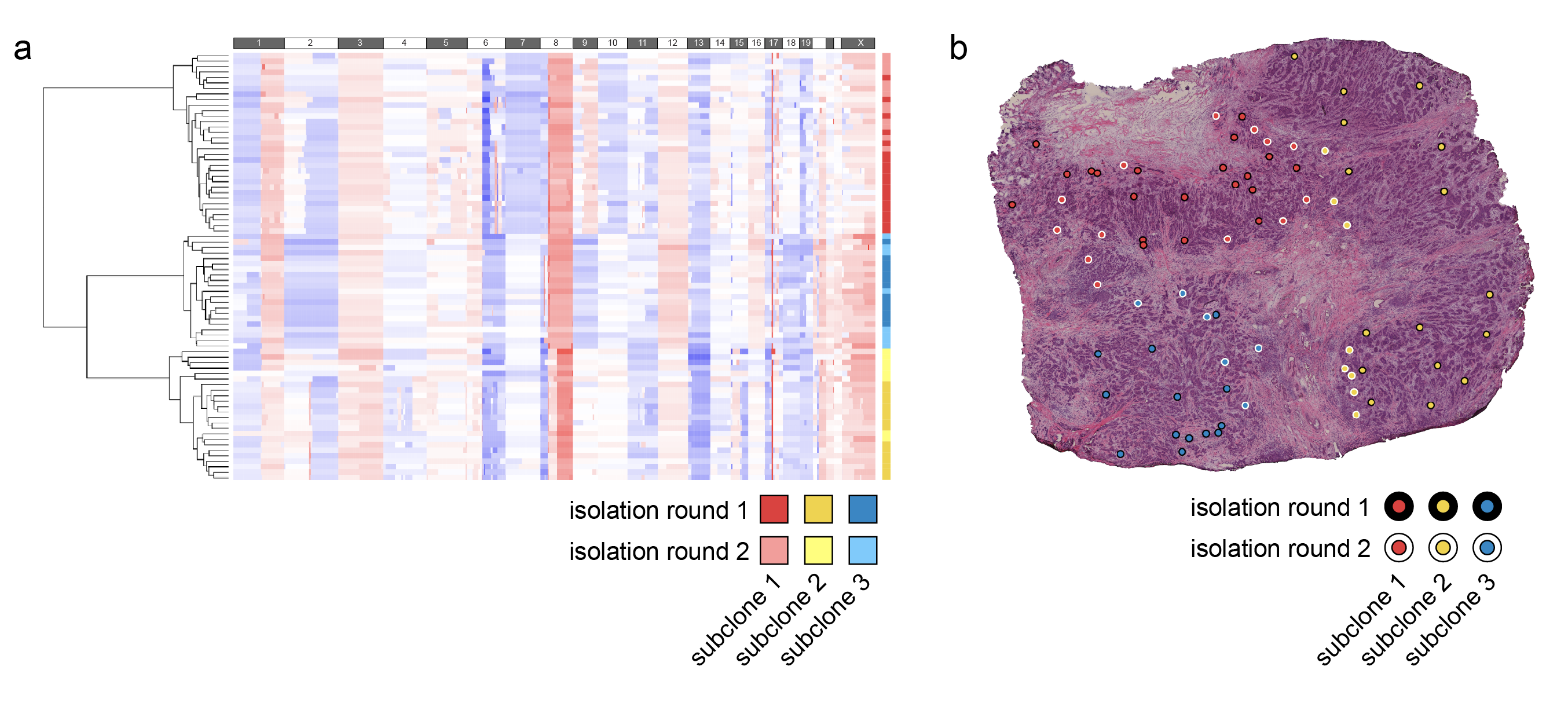
**

**Figure S6.** The evolution of the three subclones and their spatial organization is inferred through PHLI-seq. Alterations shared by the three subclones may have led to the early stage of tumorigenesis. Then, subclone 2 and the ancestor of subclones 1 and 3 may have divided and accumulated unshared alterations.

**
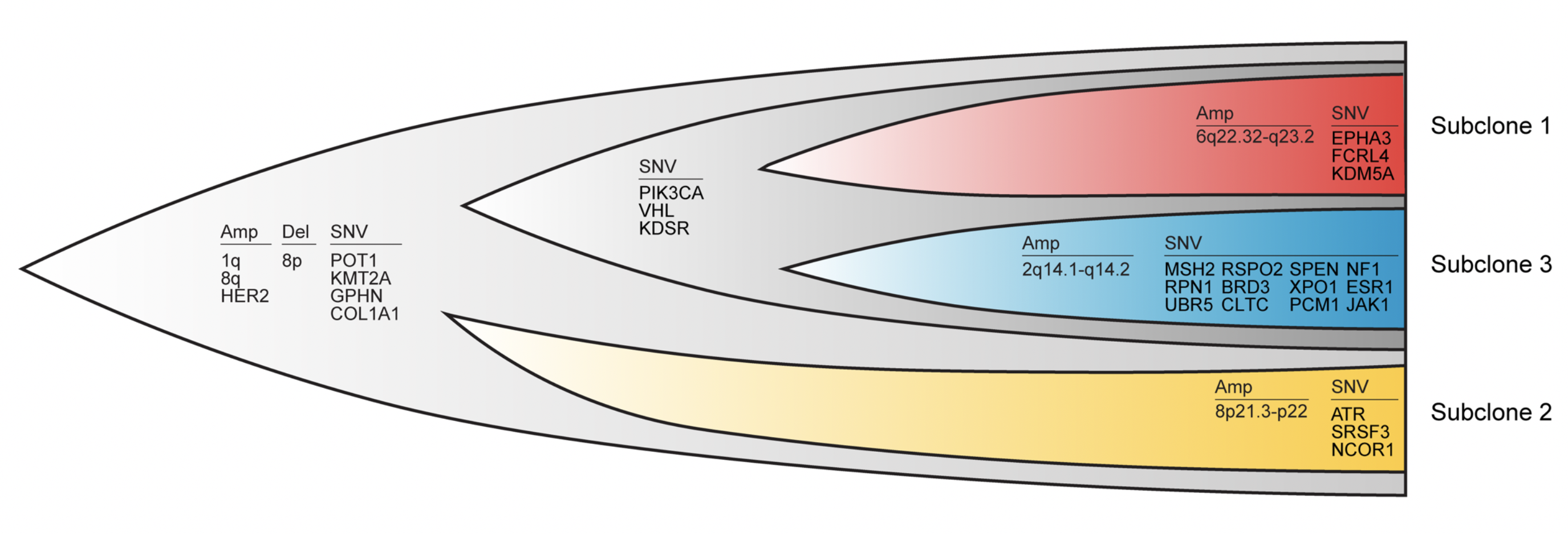
**

**Figure S7.** Before applying PHLI-seq to the triple-negative breast cancer analysis, we stained tissue sections by H&E and IHC. Serial tissue sections of the sections S2, S4, and S6, which were stained by H&E in figure 5, were prepared on standard slide glasses. The serial tissue sections were stained to analyze expression level of AR, CK5/6, Ki-67, and p53. A pathologist analyzed the stained tissue sections and selected cells to be sequenced. Based on the H&E and IHC images, the pathologist selected cells in various phenotypic status. For this tumor, the three genetic subclones based on CNA (**Fig. 5;** *in situ* clones 1, *in situ* clones 2, and invasive clone) are associated with cancer cells’ morphology in H&E tissue sections and CK5/6 expression, but not with AR, P53, and Ki-67 expression.

**
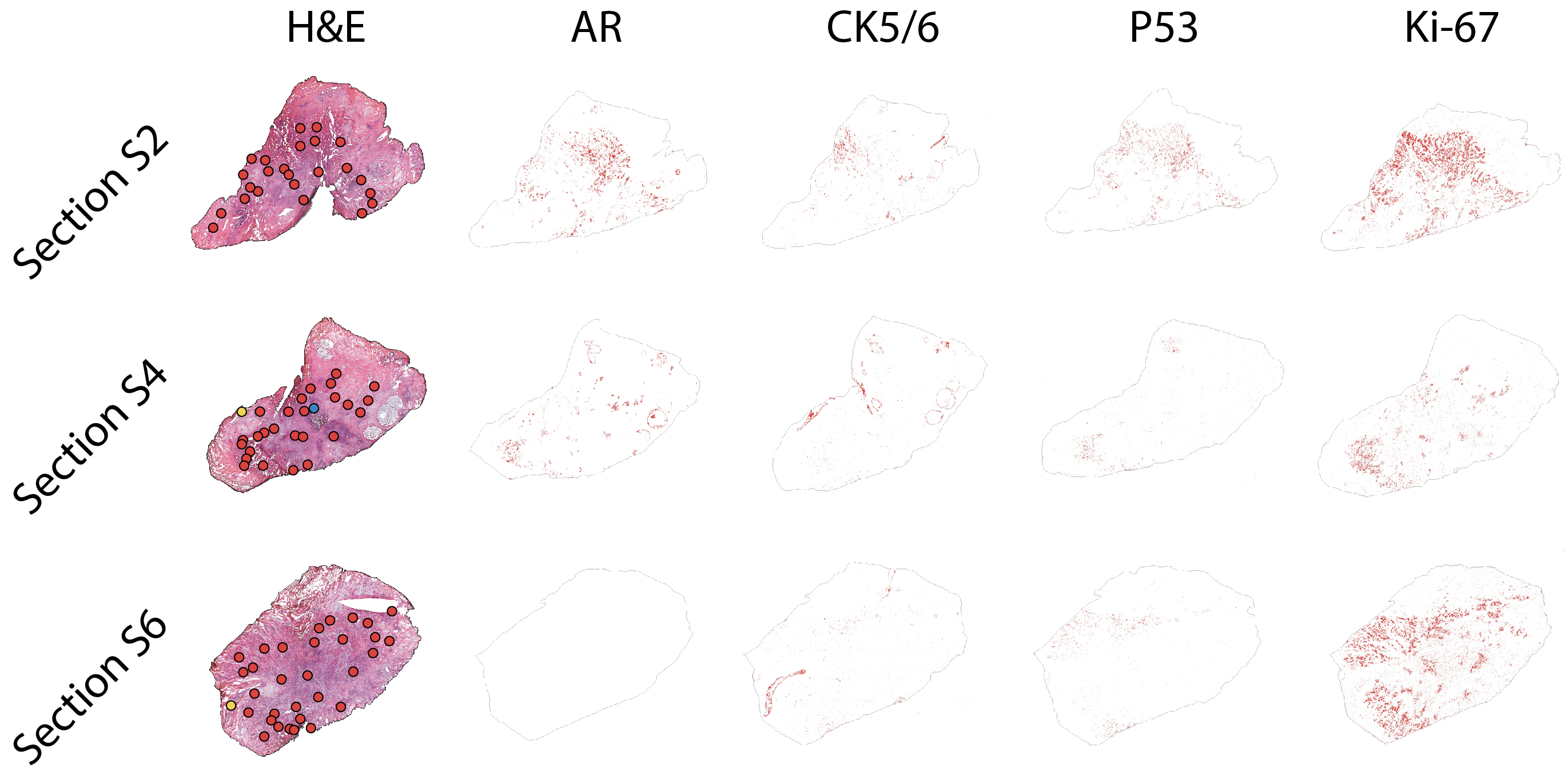
**

**
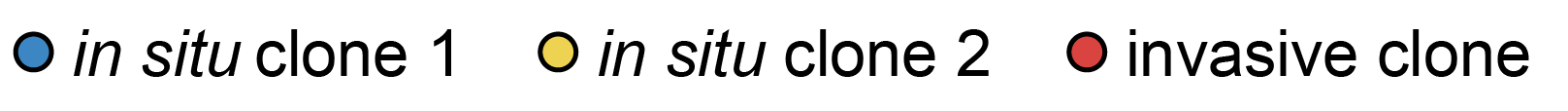
**

**Figure S8.** Finding optimal classification of cell clusters by weighted hierarchical clustering. To achieve this, we set a score function and calculated the score for various combinations of weights for 'position', ‘angle’, ‘major axis over minor axis’, and ‘cluster area’ (see Online Methods). We fixed weight of the ‘position’ as 1, and swept values for the other 3 features. We could find the highest score when weights for ‘angle’, ‘major axis over minor axis’, and ‘cluster area’ were 1/6, 1/4.5, and 1/6. (a) 3D heatmap of 36 x 36 x 36 score matrix. Each small box indicates log scaled score from a weights combination (scaled down the matrix to 18x18x18 for visualization). (b) 2D heatmap of scores by taking the maximum value from each 1D bar in (a). (c) 2D heatmap of scores as in (b), but not log scaled.


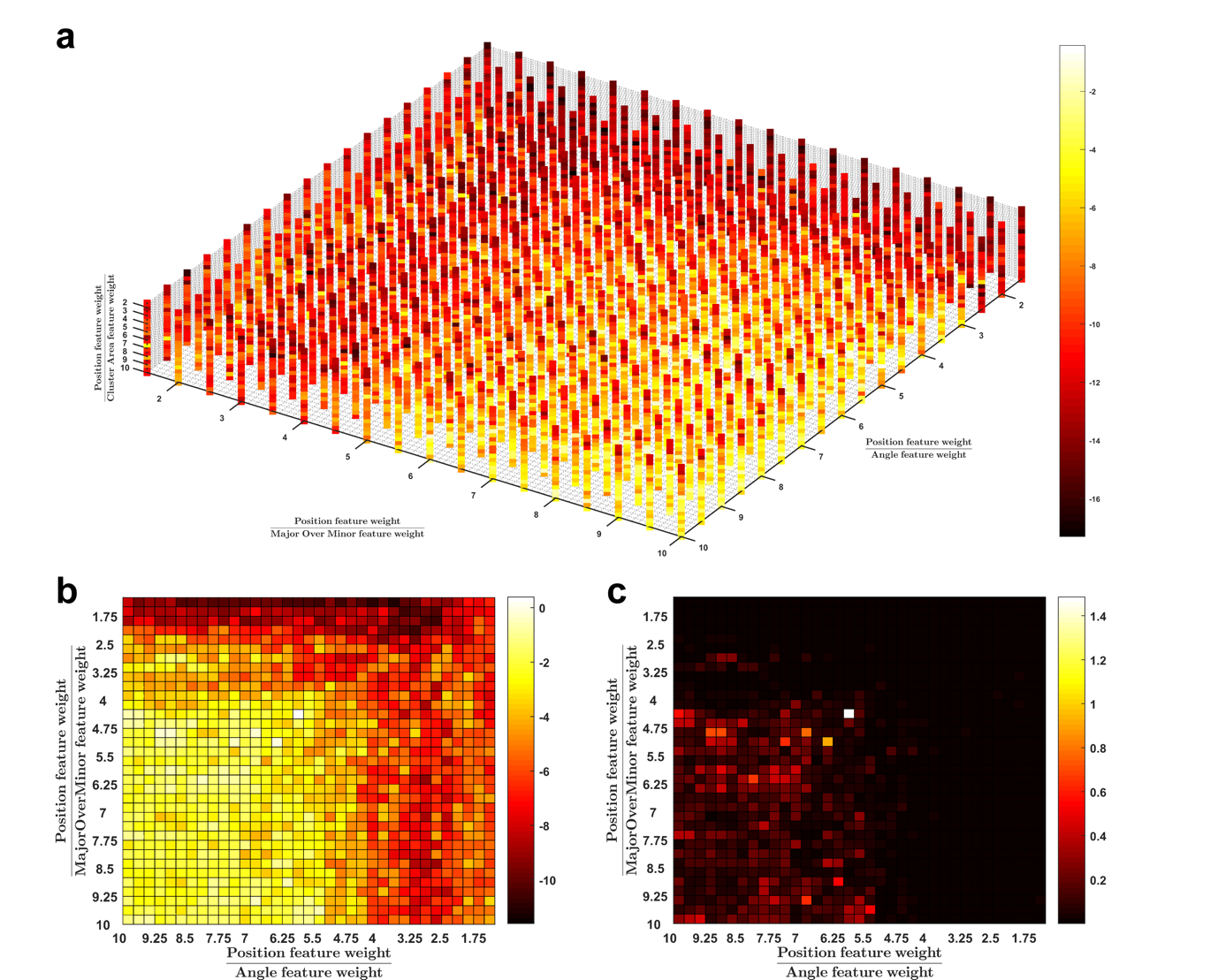


**Figure S9.** Effect of laser wavelength and discharging layer. We prepared cells or tissue on a standard glass, polymer membrane for laser capture microdissection, and discharging layer (indium tin oxide). When we applied UV laser to the samples with the energy for cutting laser microdissection membrane, we observed burning signature of tissue and cells. In contrast, when IR laser was irradiated, only cells on discharging layer were isolated without any sign of damage. In the case of glass and polymer membrane, there was no change between before and after of laser irradiation, which means IR laser transfers its energy only to discharging layer, but not to tissue and cell itself.


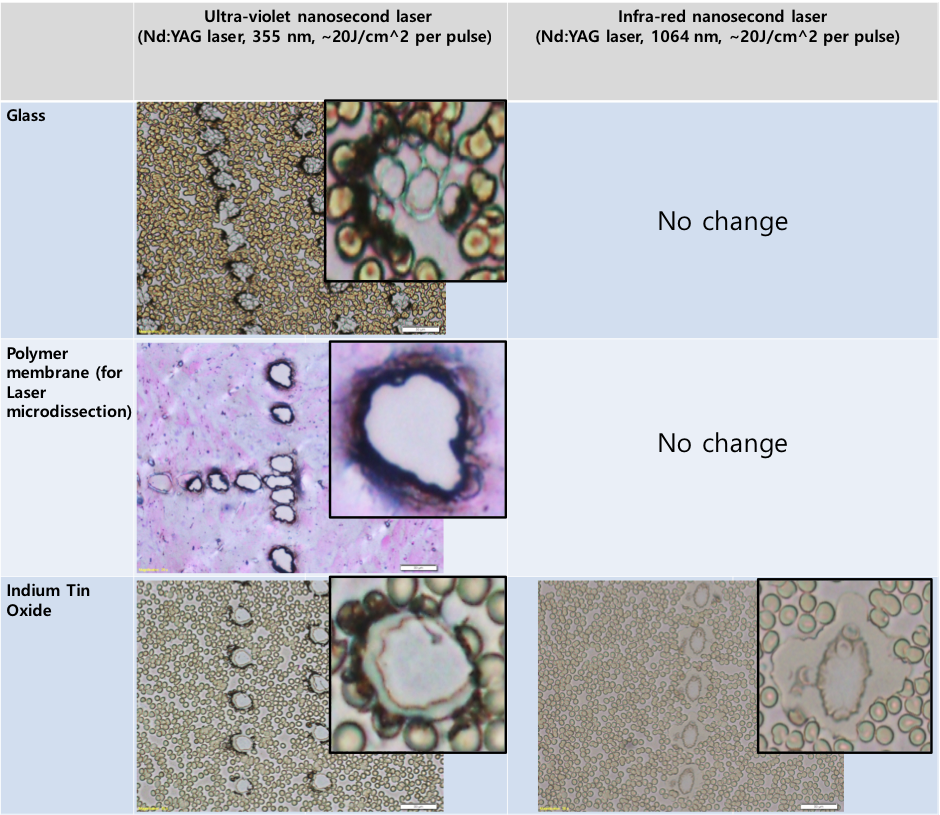


**Figure S10.** Effect of laser power on vaporizing ITO layer. We used about 5 J/cm^2^ for isolating cells in this paper. The following figure demonstrates vaporizing indium tin oxide (ITO) layer with four different laser powers. The laser powers were measured using laser power meter at the position where cells were located. The ITO layer clearly vaporized at 4 J/cm^2^.


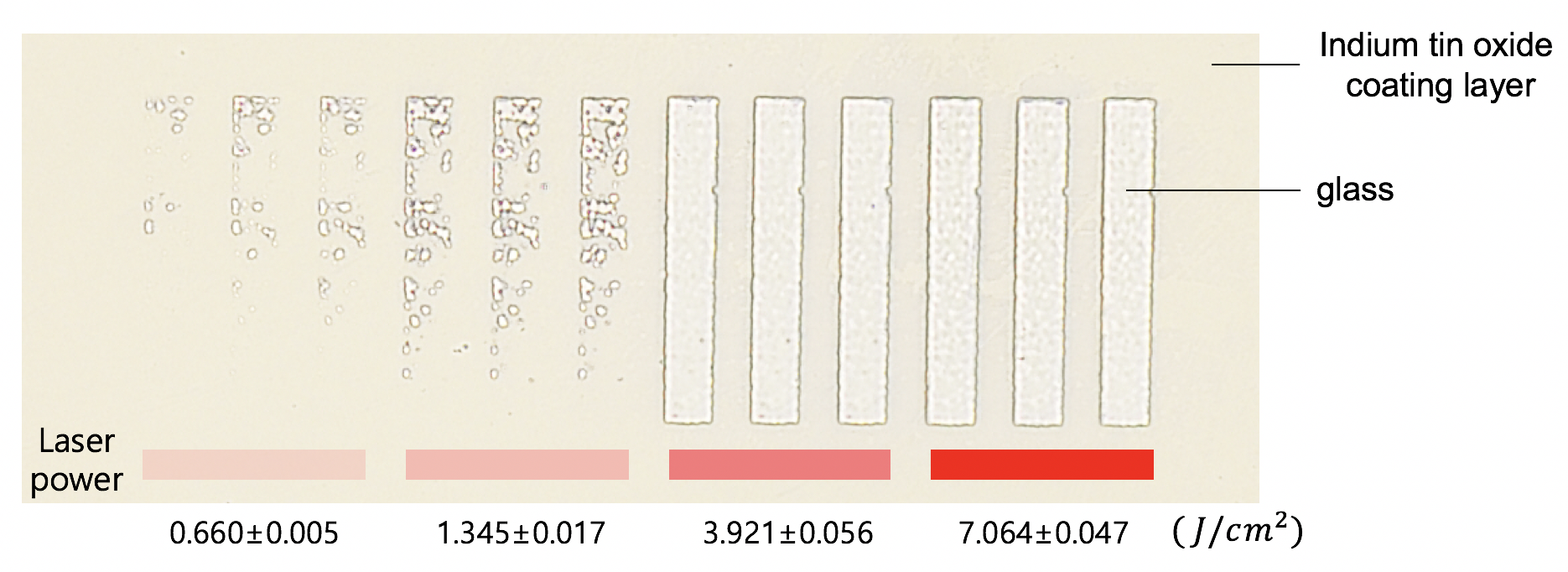


**Figure S11.** Single molecule deep sequencing library preparation and sequencing result: (a) Bioanalyzer electropherogram after ligating molecular tags to PCR library; (b) DNA molecules in 200 bp to 1000 bp were purified to remove tag dimers. Majority of DNA molecules in this product doesn’t have molecular tags at both ends. Therefore, the quantity of molecules having molecular tags at both ends should be quantified to proceed to the next step; (c-f) the ligated products were 1, 4, 16, and 160-fold diluted and PCR amplified by 10, 12, 14, and 17 cycles; (g) The amplified products were quantified to calculate original library concentration; (h) by extrapolating expected original library concentration according to PCR cycle (or dilution factor), we concluded that the concentration of properly tag-ligated molecules was ~12 amol/ul. With this number, we carried out single molecule deep sequencing; (i) from the sequencing result, we called single strand consensus sequence (SSCS), and validated SNVs detected by PHLI-seq. SNVs detected in PHLI-seq were grouped to ‘target’, and those were not detected to ‘background’. Two groups had different allele frequency distribution, and we set true SNV threshold which guarantees Benjamini–Hochberg false discovery rate < 0.05 (horizontal line).


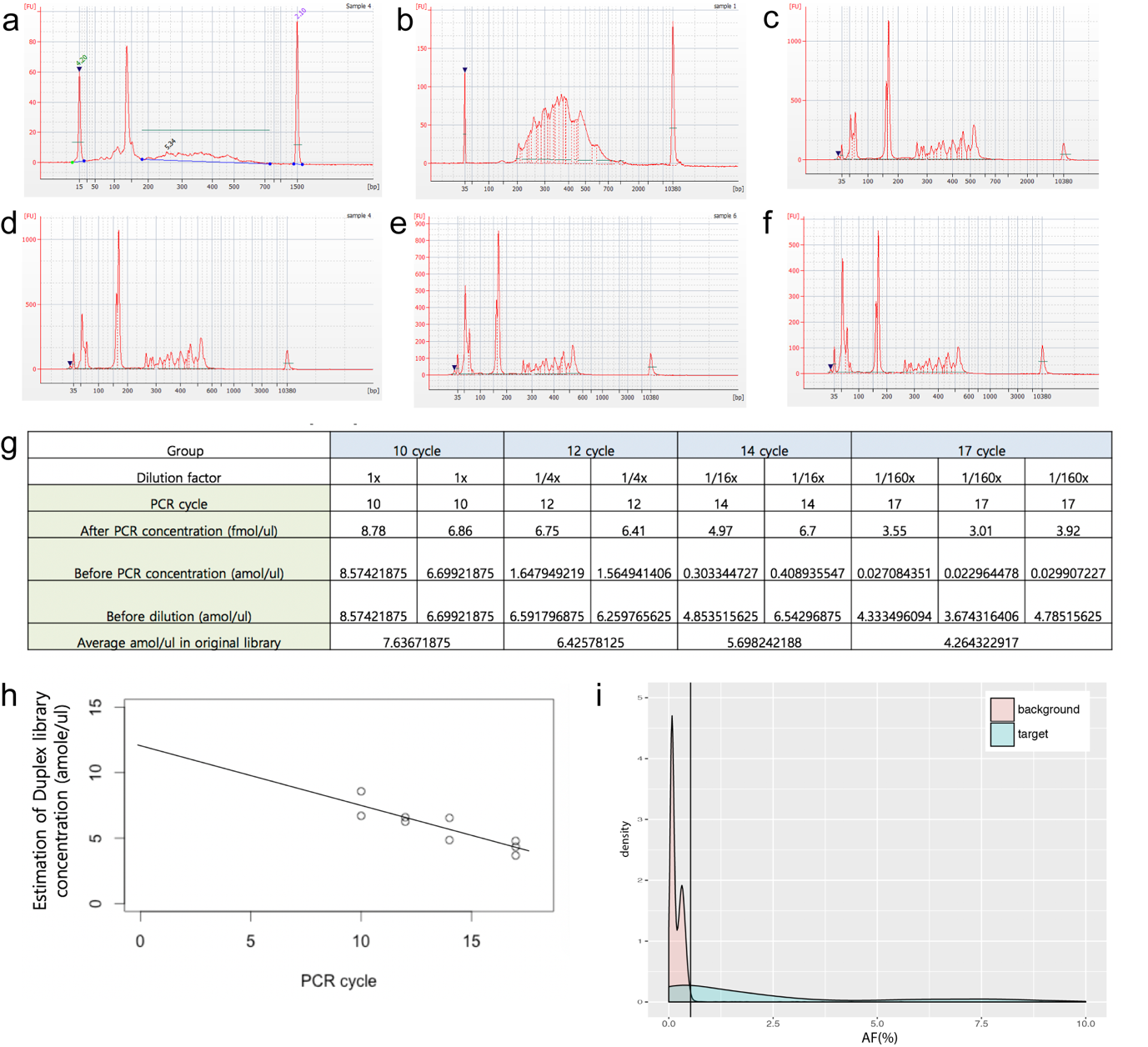


**Figure S12.** Comparison of allele dropout and false positive rate between PHLI-seq and UV-based LCM. Two serial sections from a normal tissue were prepared and stained by H&E. Then, ten cells were isolated into each tube using PHLI-seq method or UV-based LCM. The isolate cells were amplified by MDA and their whole exome were sequenced. From the whole exome sequencing data, (A) allele dropout rate and (B) false positive rate were calculated.


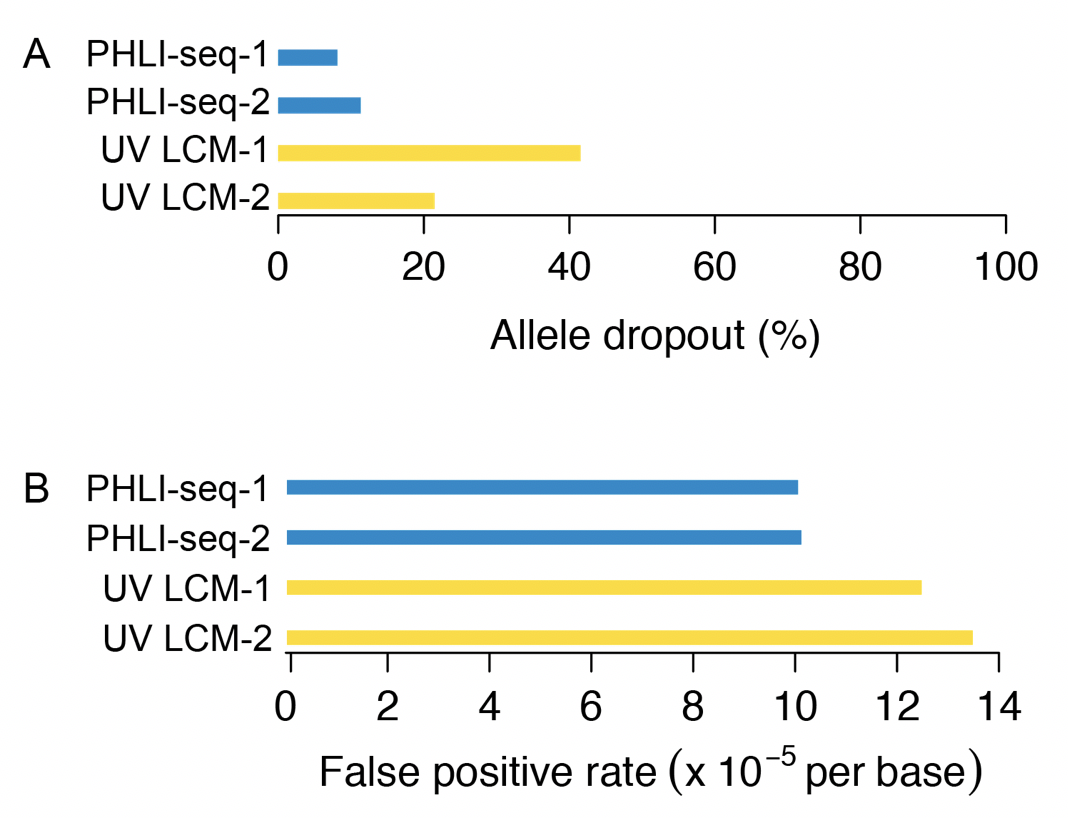


**Figure S13.** Coverage of subclone specific mutations by tumor bulk sequencing. 78.8%, 24.0%, and 6.81% of subclone 1, 2, and 3 specific mutations were covered by tumor bulk sequencing. PHLI-seq could discover subclonal mutations which were rarely covered by tumor bulk sequencing.


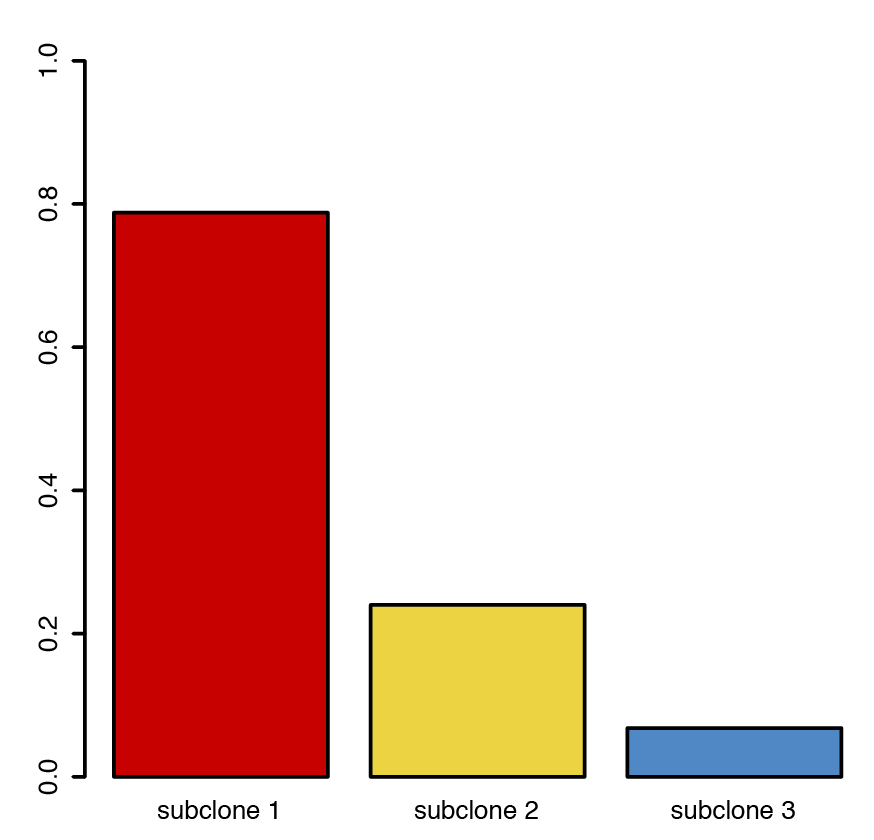


**Figure S14.** Mutation validation using single-molecule deep sequencing. We randomly selected a portion of mutations that were detected using PHLI-seq and generated a sequencing library for the targeted sites by tagging a unique molecular barcode to each DNA molecule to precisely discriminate the next-generation sequencing results from errors. Sequencing was performed to read the targeted region with an 18,182-fold single-molecule read depth. We set our validation limit to 0.55% to limit the false discovery rate to < 0.05. The results validated 92.3% (12/13) of the subclonal mutations detected by targeted sequencing. Moreover, 72.2% (78/108) of the mutations observed in the whole-exome sequencing were validated. The validation rate was 90.9% (50/55) when mutations that were observed in more than three samples were considered

**
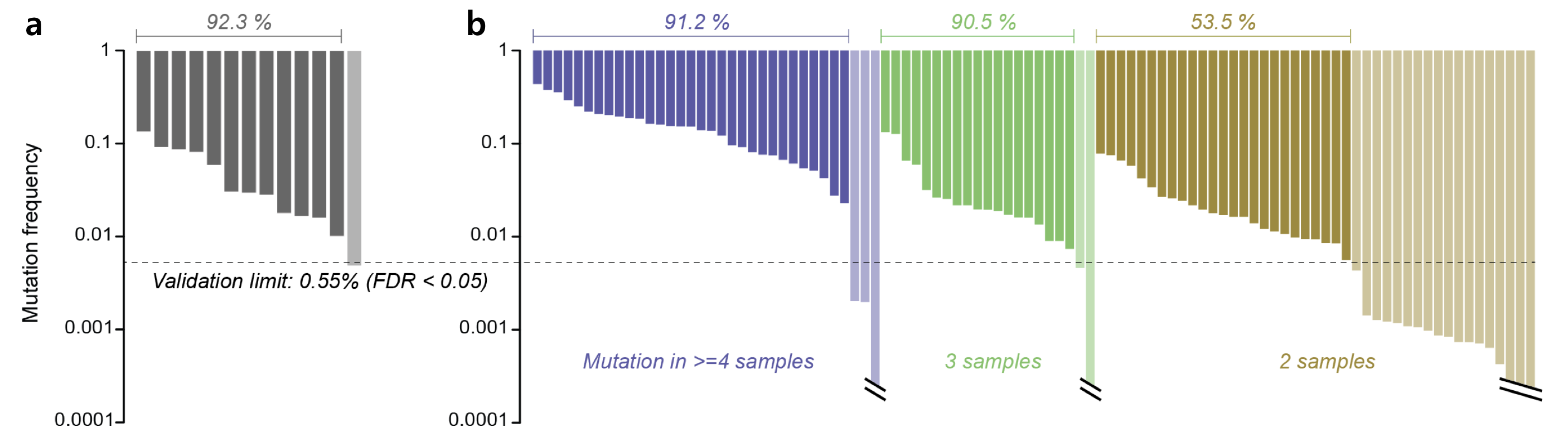
**

**Table S1.** 16 WGA validation primers list

| Validation primer 1 | F | 5'-TCTAGACCTGCCACTGGGAA-3' |
| --- | --- | --- |
|  | R | 5'-ATGCAGCAGGTGCTGAGTAA-3' |
| Validation primer 2 | F | 5'-ACTGCCCATGCACTTTGACT-3' |
|  | R | 5'-CCACACTCCTTCGCCAACTT-3' |
| Validation primer 3 | F | 5'-ACACCATGAAGCAGAAGGGG-3' |
|  | R | 5'-TGCATGAGCCCATGTACCTC-3' |
| Validation primer 4 | F | 5'-GGATGACTGGAGCAGGGAAG-3' |
|  | R | 5'-TGGGCAGCATCCATTGAGAG-3' |
| Validation primer 5 | F | 5'-AAGAGCATTTTTATGCTCCATCTG-3' |
|  | R | 5'-CACATACAGACCCGCTGGAA-3' |
| Validation primer 6 | F | 5'-GCCAACATGGCCAGGAAGTA-3' |
|  | R | 5'-TCATGTGCACAAATGTATGTTTCTT-3' |
| Validation primer 7 | F | 5'-GGAAGGCTTTGAAGAAGGTGAAT-3' |
|  | R | 5'-AGCACCAAAAAGGCACATACC-3' |
| Validation primer 8 | F | 5'-AACCCTCCCAATTCCAGTGC-3' |
|  | R | 5'-ACAGTTCTTTTCATCACTGCCG-3' |
| Validation primer 9 | F | 5'-GAGCCACATGAGTCTGCCAT-3' |
|  | R | 5'-AGAGCCAGGCTTTTGCTGAA-3' |
| Validation primer 10 | F | 5'-CTTCCTTGGGGACCACATCC-3' |
|  | R | 5'-CCCATCGTCTCTGCTGACAA-3' |
| Validation primer 11 | F | 5'-GTGTGCGGAAGGTACGGTTA-3' |
|  | R | 5'-TTGCTCCTGCTCAGGTCTTG-3' |
| Validation primer 12 | F | 5'-TCAATCTCCATGCCCAGGGT-3' |
|  | R | 5'-TTCACTGCCAACATTGCACG-3' |
| Validation primer 13 | F | 5'-GTGGACAGCTGACACGAGAG-3' |
|  | R | 5'-CGAGAGGCCACAGAAGTAGC-3' |
| Validation primer 14 | F | 5'-AGGTACCCGTACATACCAGGA-3' |
|  | R | 5'-TGGTGCTGGCAGGATAACAG-3' |
| Validation primer 15 | F | 5'-CTTGCTGGTCTGTCCCTCTG-3' |
|  | R | 5'-ATCCTCCCCCACCTCCTTTT-3' |
| Validation primer 16 | F | 5'-ACCTCAGCAACCTTCAAGAACT-3' |
|  | R | 5'-GGGTGTAGAATCAAACCAGCG-3' |

**Table S2.** The gene list of targeted sequencing

| MTOR | SETD2 | IGF2R | NOTCH1 | MDM2 | NCOR1 |
| --- | --- | --- | --- | --- | --- |
| EPHA2 | MST1R | EGFR | GATA3 | NAV3 | NF1 |
| ARID1A | EPHA3 | CDK6 | RET | TBX3 | CDK12 |
| PIK3R3 | POLQ | PIK3CG | PTEN | FLT3 | ERBB2 |
| JAK1 | ATR | MET | FGFR2 | BRCA2 | TOP2A |
| NOTCH2 | PIK3CA | BRAF | MEN1 | RB1 | BRCA1 |
| MCL1 | FGFR3 | EZH2 | MALAT1 | IRS2 | RPS6KB1 |
| DDR2 | PDGFRA | KMT2C | CCND1 | FOXA1 | STK11 |
| ABL2 | KIT | ZNF703 | FGF4 | MAP4K5 | INSR |
| KDM5B | INPP4B | EIF4EBP1 | FGF3 | AKT1 | TYK2 |
| IKBKE | FBXW7 | FGFR1 | C11orf30 | LTK | NOTCH3 |
| PARP1 | MAP3K1 | PRKDC | PAK1 | IDH2 | JAK3 |
| AKT3 | PIK3R1 | MYC | MRE11A | IGF1R | CCNE1 |
| ALK | APC | PTK2 | ATM | TSC2 | AKT2 |
| SF3B1 | PDGFRB | JAK2 | FOXM1 | CBFB | SRC |
| IDH1 | FGFR4 | CDKN2A | CDKN1B | CTCF | AURKA |
| ERBB4 | FLT4 | CDKN2B | KRAS | CDH1 | GNAS |
| FANCD2 | DDR1 | SYK | KMT2D | TP53 | PTK6 |
| VHL | ROS1 | TLR4 | ERBB3 | AURKB | RUNX1 |
| CTNNB1 | ESR1 | ABL1 | CDK4 | MAP2K4 | EP300 |
| AR |  |  |  |  |  |

**References**

[1] The Cancer Genome Atlas Network, “Comprehensive molecular portraits of human breast tumours.,” *Nature*, vol. 490, no. 7418, pp. 61–70, Oct. 2012.

[2] C. Kandoth *et al.*, “Mutational landscape and significance across 12 major cancer types.,” *Nature*, vol. 502, no. 7471, pp. 333–9, 2013.

[3] W. Malilas *et al.*, “Cancer upregulated gene 2, a novel oncogene, enhances migration and drug resistance of colon cancer cells via STAT1 activation,” *Int. J. Oncol.*, vol. 43, no. 4, pp. 1111–1116, 2013.

[4] T. Ikenoue *et al.*, “Functional Analysis of PIK3CA Gene Mutations in Human Colorectal Cancer,” *Cancer Res.*, vol. 61, no. 11, pp. 4562–4568, 2005.

[5] M. Gymnopoulos, M.-A. Elsliger, and P. K. Vogt, “Rare cancer-specific mutations in PIK3CA show gain of function,” *Proc. Natl. Acad. Sci.*, vol. 104, no. 13, pp. 5569–5574, 2007.

[6] G. Ciriello *et al.*, “Comprehensive Molecular Portraits of Invasive Lobular Breast Cancer,” *Cell*, vol. 163, no. 2, pp. 506–519, 2015.

[7] S. Li *et al.*, “Endocrine-Therapy-Resistant ESR1 Variants Revealed by Genomic Characterization of Breast-Cancer-Derived Xenografts,” *CellReports*, vol. 4, no. 6, pp. 1116–1130, 2013.

[8] K. Sato and K. Akimoto, “Expression Levels of KMT2C and SLC20A1 Identi fi ed by Information-theoretical Analysis Are Powerful Prognostic Biomarkers in Estrogen Receptor-positive Breast Cancer,” *Clin. Breast Cancer*, vol. 17, no. 3, pp. e135–e142, 2017.
