# Supplementary material for: PHLI-seq: constructing and visualizing cancer genomic maps in 3D by phenotype-based high-throughput laser-aided isolation and sequencing
Source: Genome Biol. 2018 Oct 8;19:158. doi: 10.1186/s13059-018-1543-9 (PMC6176506; doi:10.1186/s13059-018-1543-9)
Supplement: Supplementary file 8 — Supplementary scripts. (ZIP 15961 kb) [file 13059_2018_1543_MOESM8_ESM.zip › Additional file 5, Supplementary scripts/Instrument control SW/ReadMe.docx]

[PHLI-seq instrument control SW overview]


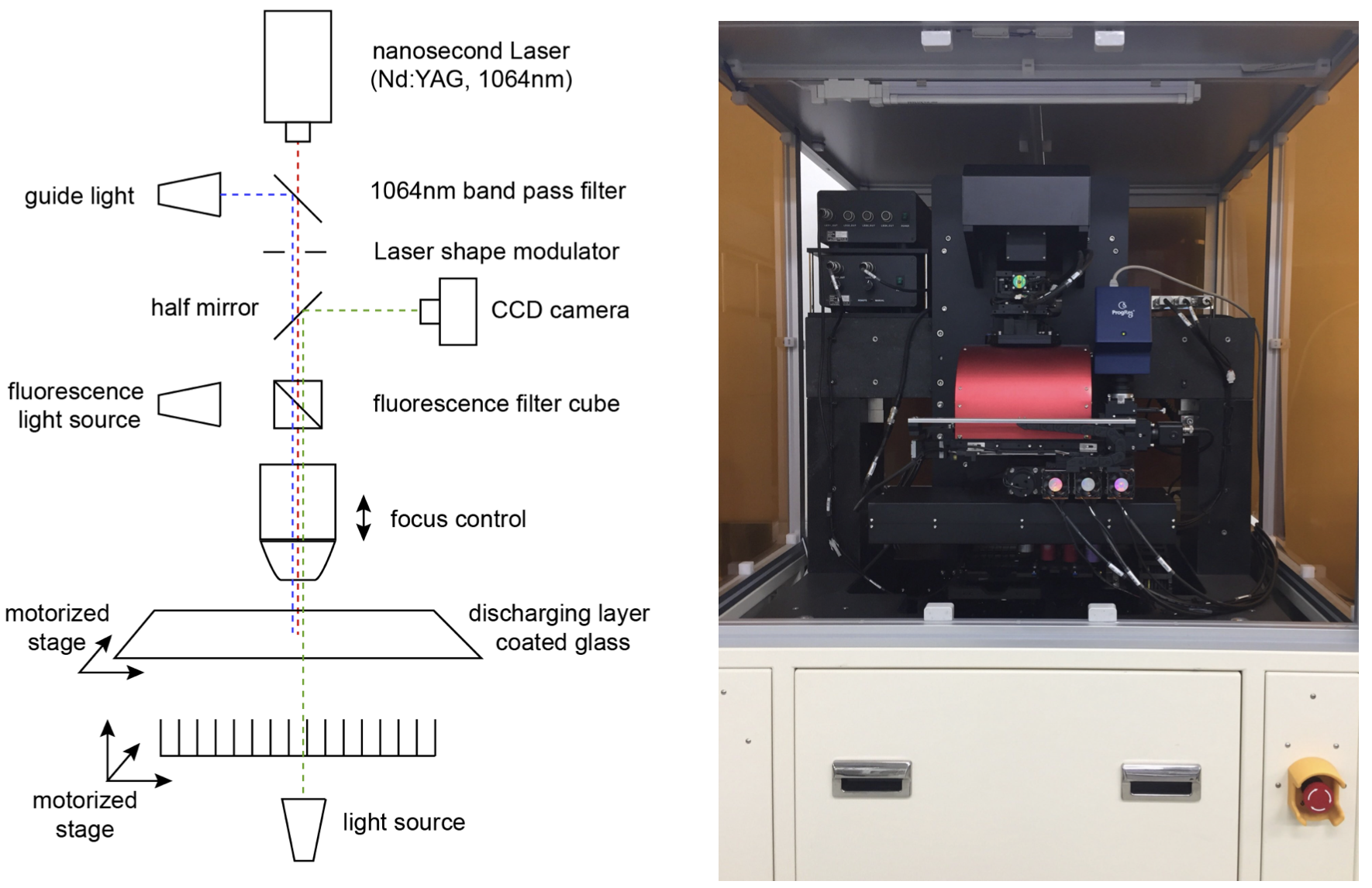


*Figure 1. Schematic and photo of PHLI-seq instrument*


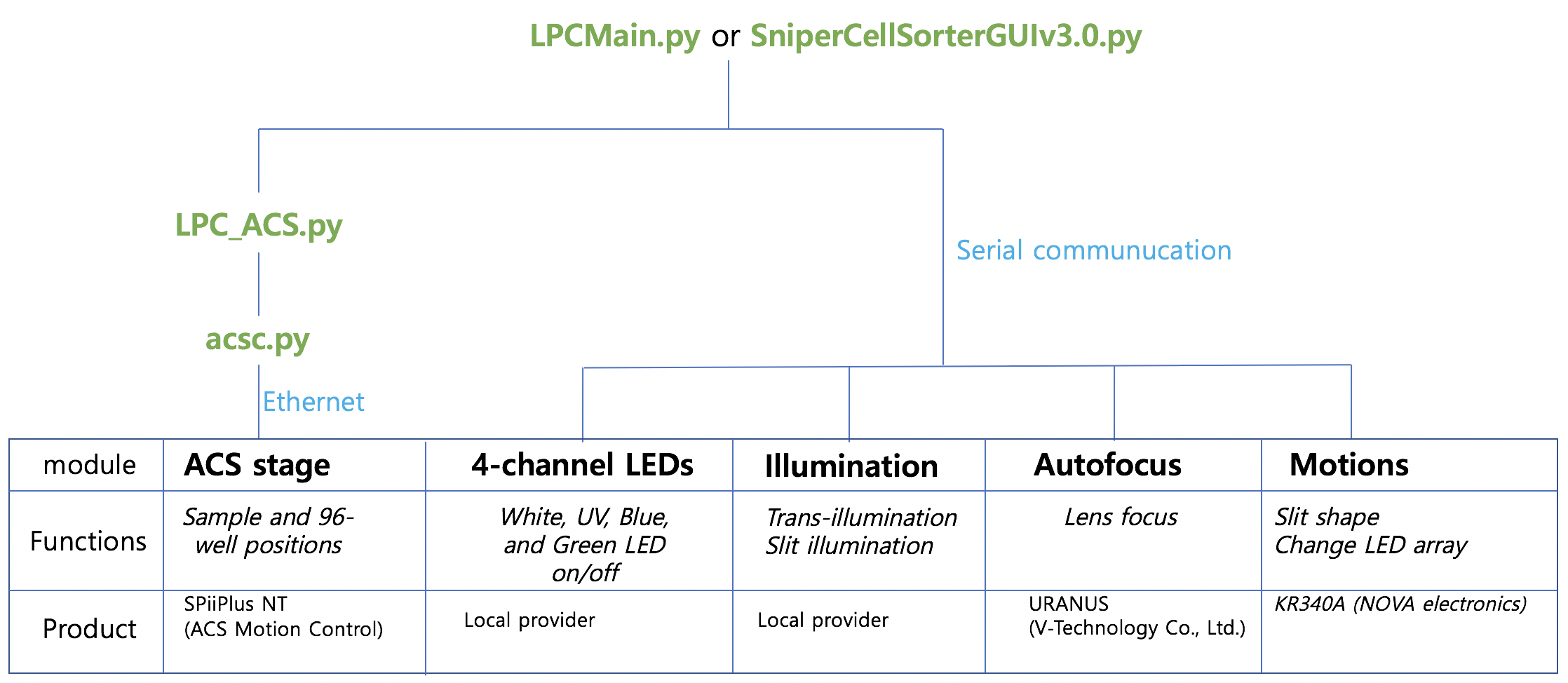


*Figure 2. The names of source codes to control the PHLI-seq instrument*

1. acsc.py: This program is a wrapper for the ACS C library using ctypes.
2. LPC_ACS.py: ACS Motorized stage operating module for PHLI-seq instrument. Ethernet communication setting depends on the local environment.
3. LPCMain.py: Interactive console to control PHLI-seq instrument. It creates Ethernet and serial communications to control every module in PHLI-seq instrument. Serial communication setting depends on local environment.
4. SniperCellSorterGUIv3.0.py: Graphical user interface (GUI) implemented version of the LPCMain.py with several additional functions. The additional functions include automatic target cell finding and isolation based on target marking files generated by SniperGUI.py.
