# Supplementary material for: PHLI-seq: constructing and visualizing cancer genomic maps in 3D by phenotype-based high-throughput laser-aided isolation and sequencing
Source: Genome Biol. 2018 Oct 8;19:158. doi: 10.1186/s13059-018-1543-9 (PMC6176506; doi:10.1186/s13059-018-1543-9)
Supplement: Supplementary file 8 — Supplementary scripts. (ZIP 15961 kb) [file 13059_2018_1543_MOESM8_ESM.zip › Additional file 5, Supplementary scripts/Cell marking SW (SniperGUI)/SniperGUI Manual.docx]

**[SniperGUI Manual]**

1. **General description of the Graphical User Interface (GUI)**This program is for marking cells to be isolated in a tissue. Marked cells can be isolated automatically using PHLI-seq instrument and SniperCellSorterGUI.py. Output files of SniperGUI.py are required in SniperCellSorterGUI.py for automatic isolation.


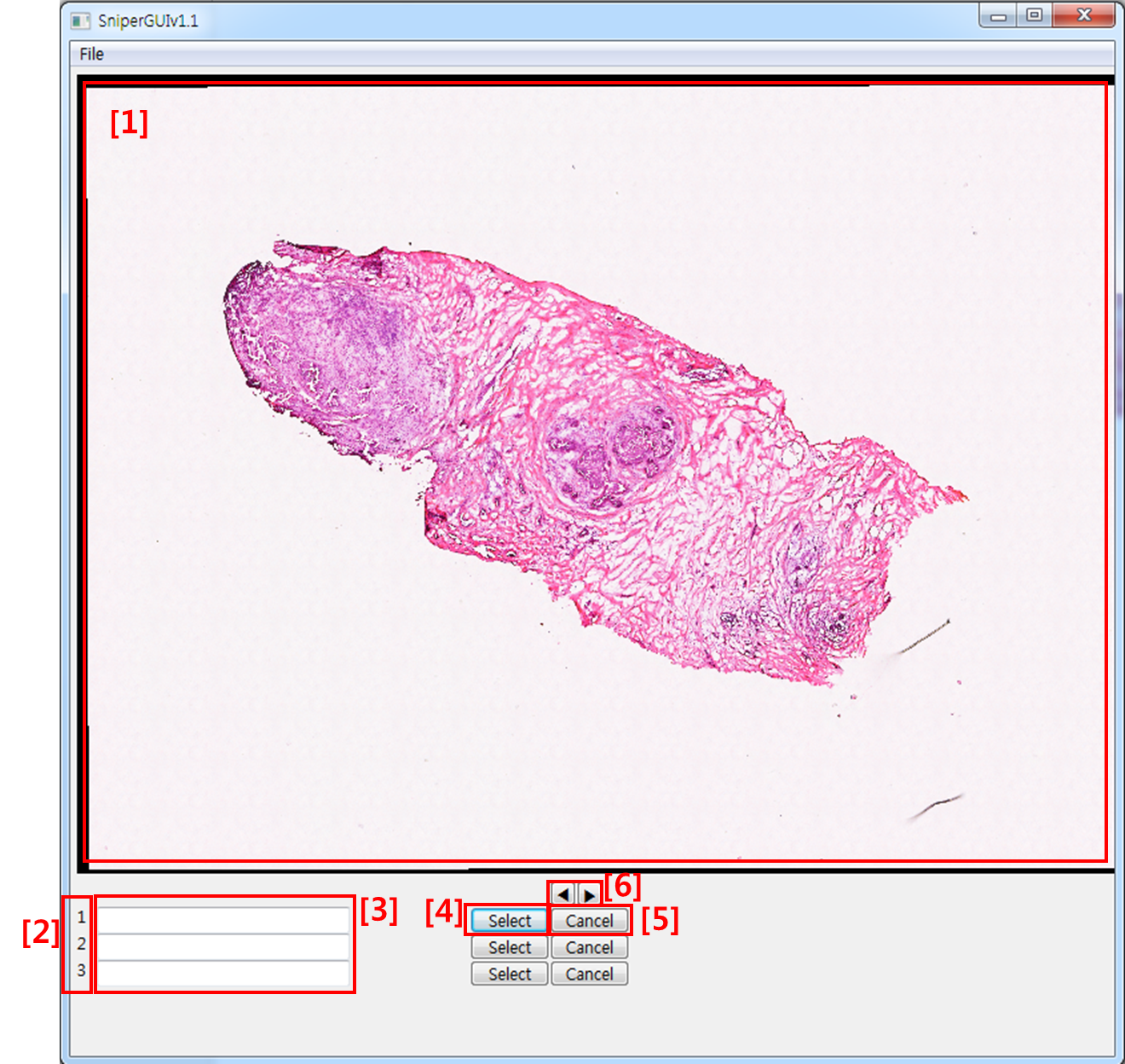


[1]: Image viewer

[2]: target index

[3]: description

[4]: target selection

[5]: cancel the selection

[6]: change index

1. **Image observation**
2. **Open image**


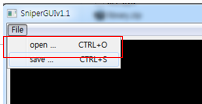


1. **Zoom in**: Ctrl + Mouse left click


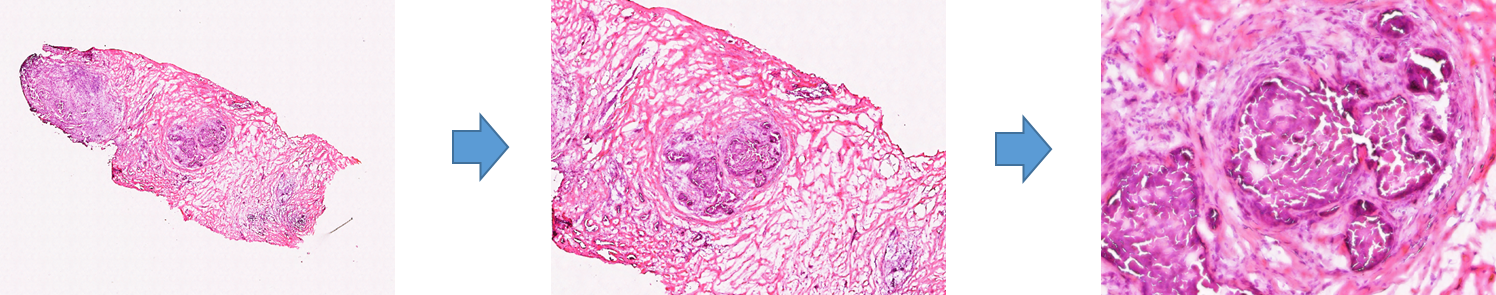


1. **Zoom out**: Ctrl + Mouse right click
2. **Move:** Mouse left click + drag
3. **Select targets**
4. **Select a region**: Mouse left click + shift + drag


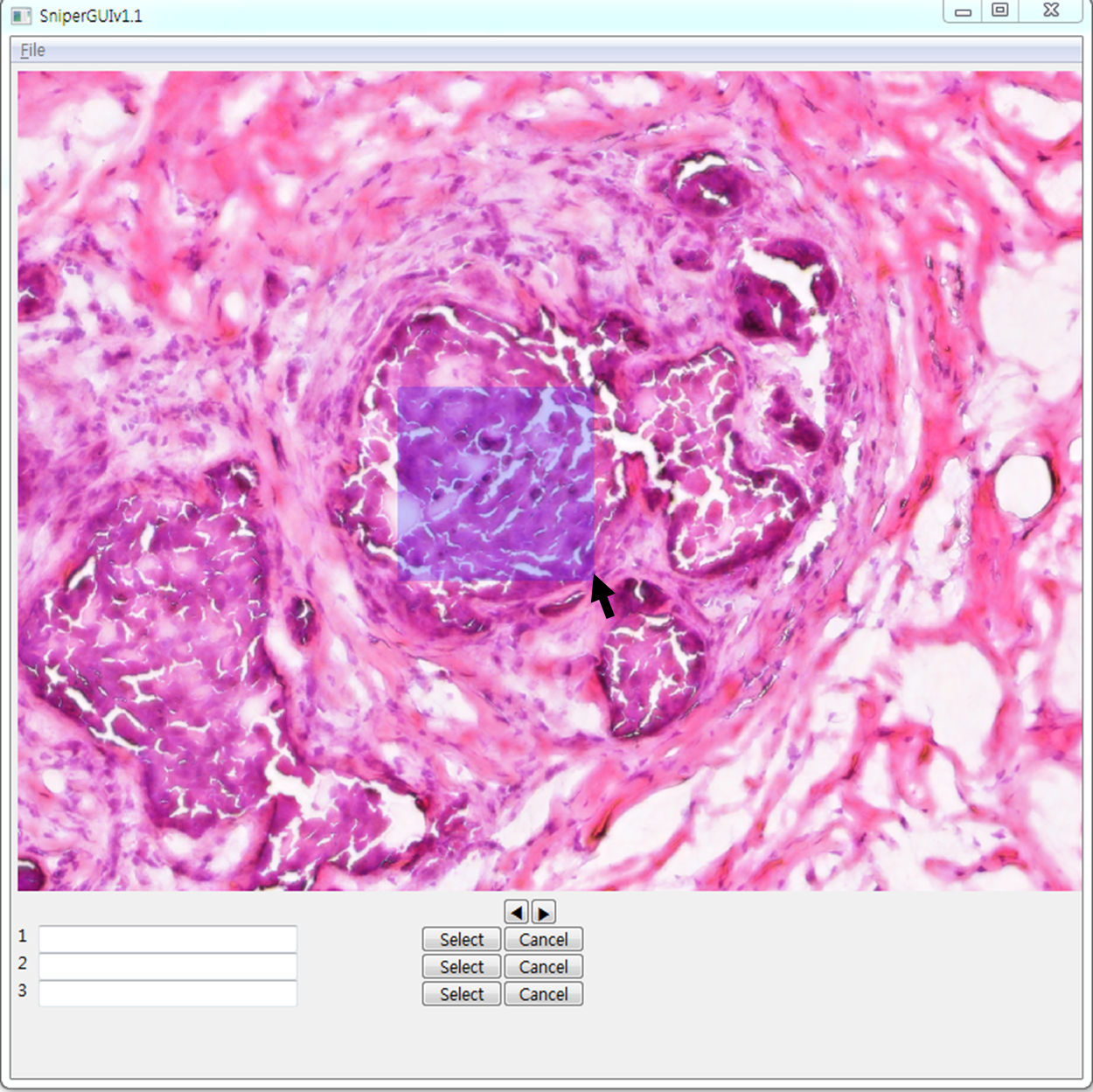


1. **Write description**


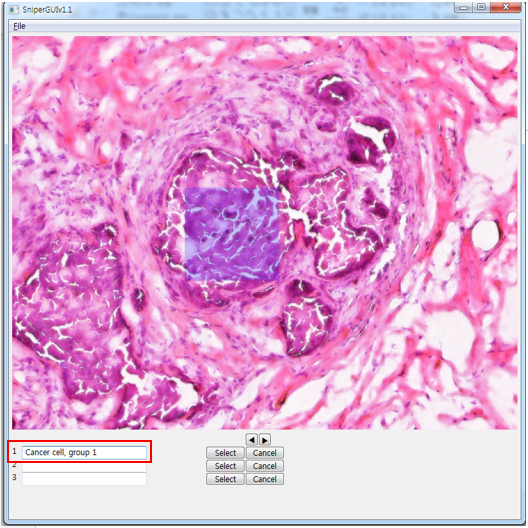


1. **Select target cells**


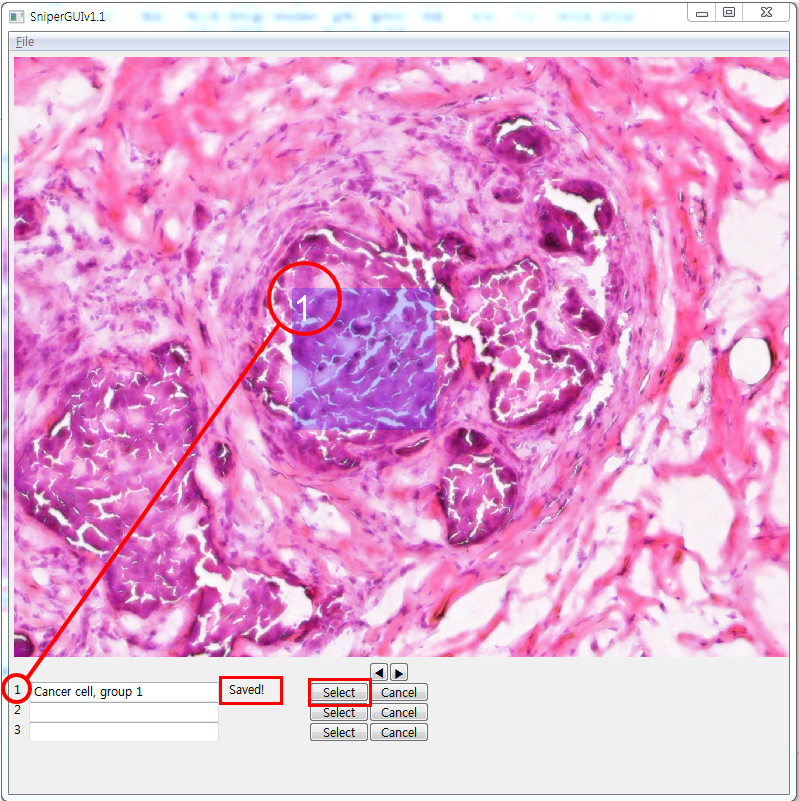


1. **Change Index**


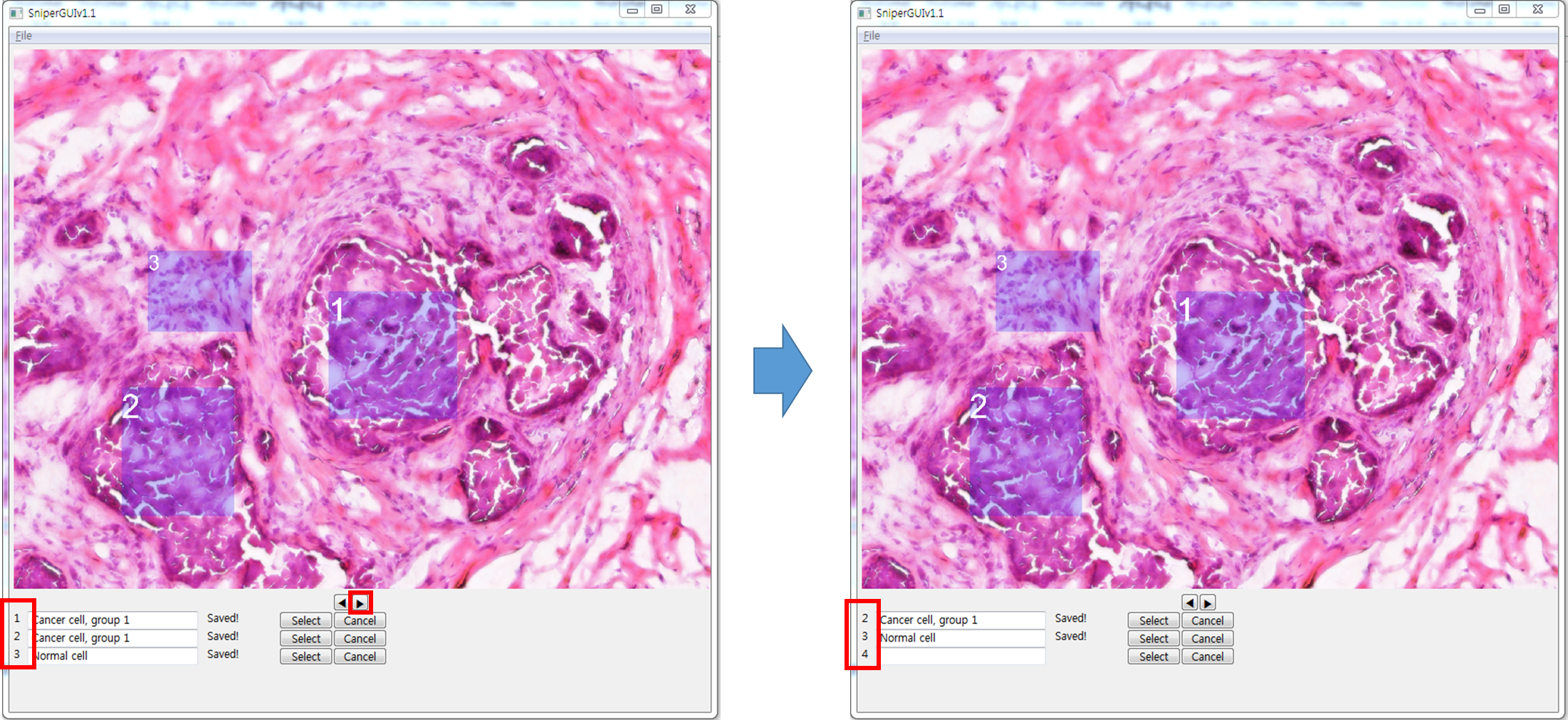


1. **Save data**
2. File > Save


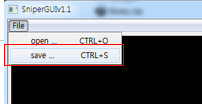


1. Output file creation

**
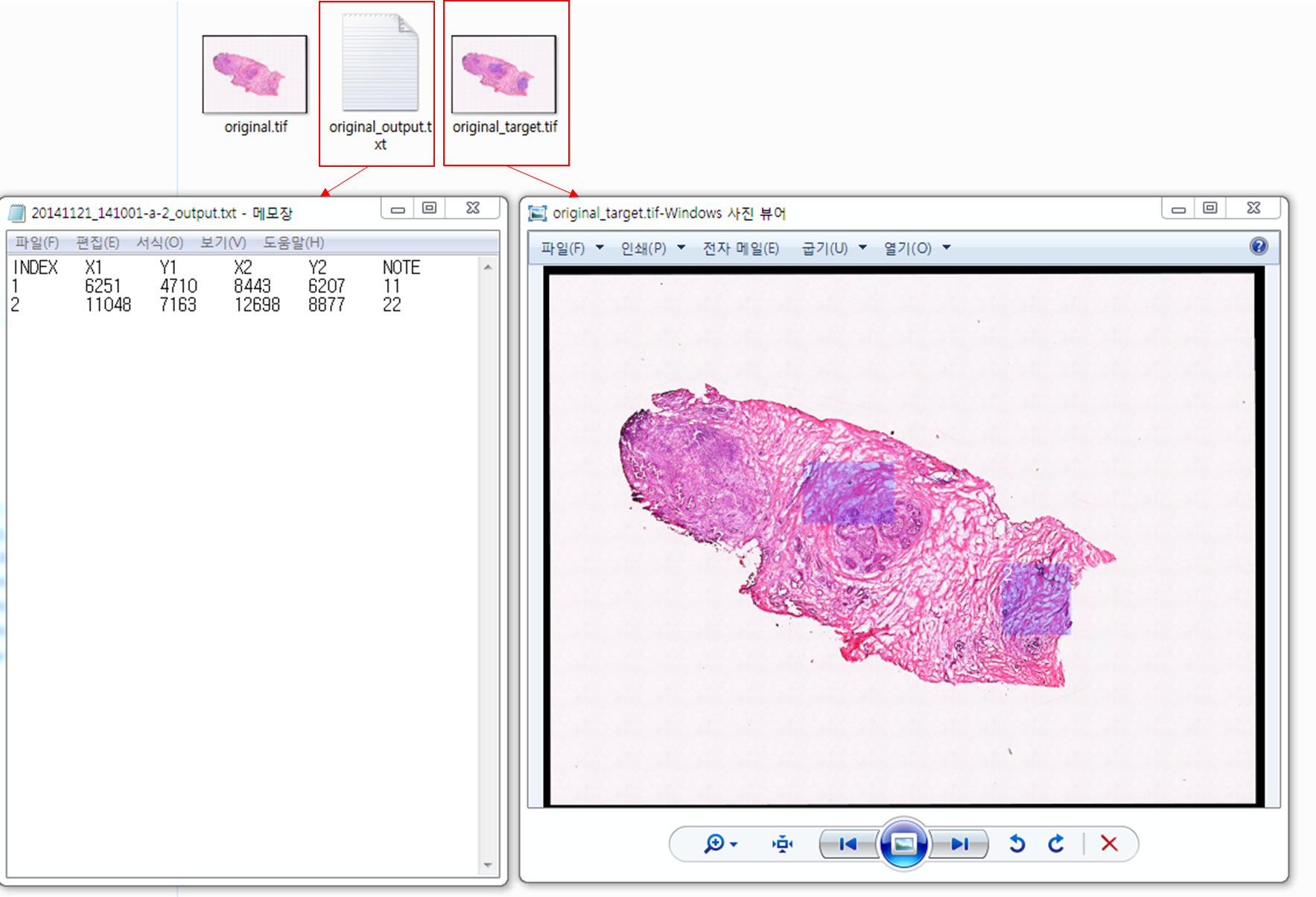
**

1. After saving, this program will generate *filename*_output.txt and *filename*_target.tif as output files. *filename*_output.txt contains locations and descriptions of the targeted cells on the image. *filename*_target.tif is an image file which will provide images of the targeted cells. These two files are required in SniperCellSorterGUI.py for automatic isolation.

Movie link: <http://youtu.be/VNk9LndnfnQ>

Questions: [amoslee89@gmail.com](mailto:amoslee89@gmail.com), 010-2700-0849
